# CPEB2-activated axonal translation of VGLUT2 mRNA promotes glutamatergic transmission and presynaptic plasticity

**DOI:** 10.1186/s12929-024-01061-2

**Published:** 2024-07-11

**Authors:** Wen-Hsin Lu, Tzu-Tung Chang, Yao-Ming Chang, Yi-Hsiang Liu, Chia-Hsuan Lin, Ching-Shu Suen, Ming-Jing Hwang, Yi-Shuian Huang

**Affiliations:** 1 Institute of Biomedical Sciences, Academia Sinica, 128 Sec. 2, Academia Rd., Taipei, 11529 Taiwan; 2 Taiwan International Graduate Program in Interdisciplinary Neuroscience, National Yang-Ming Chao-Tung University and Academia Sinica, Taipei, 11529 Taiwan; 3 Neuroscience Program of Academia Sinica, Academia Sinica, Taipei, 11529 Taiwan

**Keywords:** CPEB2, *Slc17a6*, VGLUT2, Axonal translation, Long-term potentiation, Memory, Presynaptic plasticity, Schaffer collateral pathway, Temporoammonic pathway

## Abstract

**Background:**

Local translation at synapses is important for rapidly remodeling the synaptic proteome to sustain long-term plasticity and memory. While the regulatory mechanisms underlying memory-associated local translation have been widely elucidated in the postsynaptic/dendritic region, there is no direct evidence for which RNA-binding protein (RBP) in axons controls target-specific mRNA translation to promote long-term potentiation (LTP) and memory. We previously reported that translation controlled by cytoplasmic polyadenylation element binding protein 2 (CPEB2) is important for postsynaptic plasticity and memory. Here, we investigated whether CPEB2 regulates axonal translation to support presynaptic plasticity.

**Methods:**

Behavioral and electrophysiological assessments were conducted in mice with pan neuron/glia- or glutamatergic neuron-specific knockout of CPEB2. Hippocampal Schaffer collateral (SC)-CA1 and temporoammonic (TA)-CA1 pathways were electro-recorded to monitor synaptic transmission and LTP evoked by 4 trains of high-frequency stimulation. RNA immunoprecipitation, coupled with bioinformatics analysis, were used to unveil CPEB2-binding axonal RNA candidates associated with learning, which were further validated by Western blotting and luciferase reporter assays. Adeno-associated viruses expressing Cre recombinase were stereotaxically delivered to the pre- or post-synaptic region of the TA circuit to ablate *Cpeb2* for further electrophysiological investigation. Biochemically isolated synaptosomes and axotomized neurons cultured on a microfluidic platform were applied to measure axonal protein synthesis and FM4-64FX-loaded synaptic vesicles.

**Results:**

Electrophysiological analysis of hippocampal CA1 neurons detected abnormal excitability and vesicle release probability in CPEB2-depleted SC and TA afferents, so we cross-compared the CPEB2-immunoprecipitated transcriptome with a learning-induced axonal translatome in the adult cortex to identify axonal targets possibly regulated by CPEB2. We validated that *Slc17a6*, encoding vesicular glutamate transporter 2 (VGLUT2), is translationally upregulated by CPEB2. Conditional knockout of CPEB2 in VGLUT2-expressing glutamatergic neurons impaired consolidation of hippocampus-dependent memory in mice. Presynaptic-specific ablation of *Cpeb2* in VGLUT2-dominated TA afferents was sufficient to attenuate protein synthesis-dependent LTP. Moreover, blocking activity-induced axonal *Slc17a6* translation by CPEB2 deficiency or cycloheximide diminished the releasable pool of VGLUT2-containing synaptic vesicles.

**Conclusions:**

We identified 272 CPEB2-binding transcripts with altered axonal translation post-learning and established a causal link between CPEB2-driven axonal synthesis of VGLUT2 and presynaptic translation-dependent LTP. These findings extend our understanding of memory-related translational control mechanisms in the presynaptic compartment.

**Supplementary Information:**

The online version contains supplementary material available at 10.1186/s12929-024-01061-2.

## Background

Encoding transient learned information into long-term memory requires de novo protein synthesis to maintain long-lasting modifications of activated synapses [1]. To achieve input-specific reorganization of the proteome only at participating synapses for strengthening memory engrams, local translation is a more effective means than long-distance convoy of proteins from the soma, given that axonal terminals can be, to the extreme, a meter away from soma [2–5]. Nevertheless, the study of local translation mechanisms has focused on postsynaptic dendrites because in electron micrographs of the adult mammalian brain, no polyribosome was confidently observed at presynaptic axonal terminals as compared with postsynaptic dendritic shafts [6]. In contrast, regulated translation has been found in invertebrate axons [7, 8], growth cones of developing central nervous system axons [9–12], and regenerating dorsal root ganglion axons [13–16]. Several axonal transcriptomes and proteomes from cultured neurons, developing retinal ganglia, and developing callosal projection of the cortex reinforced the existence of a unique repertoire of mRNAs in axons [9, 10, 17, 18].

Although axonal translation was historically thought to exist only in developing and regenerating mammalian axons, recent biochemical and imaging studies have detected ribosomes in mature axons, some of which exist in monosomes [19–21]. Likewise, despite the lack of axonal ultrastructure like endoplasmic reticulum and Golgi apparatus, mRNAs encoding transmembrane and secretory proteins were found in axons [17, 18, 20, 22] and membrane-targeting and secretion of axonal proteins could be blocked locally by brefeldin A [23, 24], showing the presence of functional endoplasmic reticulum and Golgi routes in axons. Moreover, cannabinoid retrograde signaling increased presynaptic protein synthesis to support specific forms of long-term depression in hippocampal and striatal slices prepared from juvenile rodents [21, 25]. Importantly, a study using the translating ribosome affinity purification (TRAP) technique first profiled a learning-associated axonal translatome in cortico-amygdala projections (i.e., TE3 cortical region to lateral amygdala) of cued fear-conditioned adult rats [20]. Although these studies confirm that axonal protein synthesis in mature neurons is associated with synaptic plasticity and modulated by the animal’s learning experience, there is no direct evidence for which RBP in axons controls target-specific mRNA translation to regulate LTP and memory. To bridge this gap, we investigated the presynaptic role of CPEB2.

CPEB2 belongs to the CPEB family of 4 proteins in vertebrates, all of which bind to the CPE (UUUUA_1-2_U) and control translation of target-specific mRNAs in neurons [26–35]. Although most identified CPEB-regulated mRNAs function at postsynaptic sites, CPEB1-mediated translation in *Aplysia* axons and growth cones of developing hippocampal neurons is essential for the persistence of long-term facilitation [7, 8] and the growth and branching of processes [36], respectively. The loss of CPEB1, CPEB3 or CPEB4 in the presynaptic CA3 neurons in the SC-CA1 pathway did not affect paired-pulse facilitation (PPF) [26, 37, 38], a short form of presynaptic plasticity as a transient increase in the probability of vesicle release [39]. Because most CPEB2-knockout (KO) mice die within 3 days after birth due to respiratory failure [40, 41], the SC-CA1 circuit was only investigated in CPEB2-conditional KO (cKO^Camk2^) mice whose *Cpeb2* was ablated in postsynaptic CA1 but not presynaptic CA3 neurons [35]. CPEB2-promoted postsynaptic translation of GRIP-associated protein 1 (GRASP1) mRNA is essential to control the surface expression of alpha-amino-3-hydroxy-5-methyl-4-isoxazolepropionic acid-type glutamate receptors (AMPARs) to support long-lasting LTP and spatial memory [35]. However, the role of CPEB2 in presynaptic translation and plasticity has not been investigated.

In this study, we identified that CPEB2 deficiency in presynaptic neurons reduced fiber volley amplitude, enhanced PPF, and impaired protein synthesis-dependent LTP in both SC-CA1 and TA-CA1 pathways. Cross-comparison of the CPEB2-immunoprecipitated transcriptome with the learning-associated axonal translatome [20] revealed *Slc17a6*, whose expression was translationally upregulated by CPEB2. *Slc17a6* encodes VGLUT2, a presynaptic protein that takes up glutamate into synaptic vesicles [42–44]. Moreover, high-frequency stimulation induced CPEB2-mediated translation in transected VGLUT2-positive (VGLUT2^+^) axons, which was sufficient to support protein synthesis-dependent LTP in the TA-CA1 pathway and replenish the releasable pool of VGLUT2^+^ vesicles in cultured neurons. Together with our previous findings [35], CPEB2 regulates not only post- but also pre-synaptic translation to coordinately strengthen glutamatergic circuits, thereby leading to long-term modifications in behavior.

## Methods

### Animals and genotyping

All procedures were approved by Institutional Animal Care and Use Committee (protocol number: 12–12-491). Mice were housed in a 12-h light–dark (lights on from 8:00 to 20:00) room with ad libitum access to food and water. *Nestin*-Cre (JAX#003771) and *Vglut2*-Cre (JAX#028863) mice were from the Jackson Laboratory [45, 46]. CPEB2-cWT and -cKO mice from the mating of *Cpeb2*^f/f, *Nestin*−Cre/+^ (cKO^Nes^) or *Cpeb2*^f/f, *Vglut2*−Cre/+^ (cKO^Vglut2^) male and *Cpeb2*^f/f, +/+^ (cWT) female mice were used for the study. CPEB2-WT and -KO embryos or adult mice were collected from *Cpeb2*^+/-^ heterozygous mating. The genotypes were determined by PCR of tail biopsies as described [41].

### Intracranial injection of adeno-associated virus (AAV)

Recombinant AAV viruses, ssAAV9-Cre-ires-GFP (5.4 × 10^10^ vg/μl, GFP expression is mediated by internal ribosome entry site), dsAAV8-GFP (1.4 × 10^10^ vg/μl) and ssAAV9-GFP (5.4 × 10^10^ vg/μl), were purchased from the institutional AAV core. Stereotaxic injection was applied to male mice as previously mentioned with modifications [35]. In brief, the entorhinal cortex (EC) region (AP—2.46 mm, ML ± 4.29 mm, DV—4.25 mm relative to bregma) and CA1 region (AP—2.00 mm, ML ± 1.25 mm, DV—1.20 mm relative to bregma) were located by using the Robot Stereotaxic system (Neurostar). A 33-gauge needle syringe was used to deliver 0.3 μl virus to each region at the rate of 12 μl/h. After surgery, the mice were monitored until they were fully ambulatory and then recovered for at least 4 weeks in housing cages before field recording.

### Behavioral assays and electrophysiology

All behavioral assays and field recording in the SC-CA1 circuit were previously described [26, 35] (Additional File 1: Supplemental method). To avoid the influence of estrous cycle in behavioral performance, only male littermates between 2 and 4 months old were used for behavioral tasks during a 13:30–17:30 light phase with the observer blinded to their genotypes. Littermates of both sexes were used for SC-CA1 recording, but only male mice were used for TA-CA1 recording because sex differences were observed in the latter circuit [47]. The electrophysiological responses were recorded at stratum radiatum (SC pathway) or stratum lacunosum-moleculare (TA pathway) of CA1 neurons after SC axons and TA axons were severed from the soma of CA3 and entorhinal cortical neurons, respectively [47, 48]. The input–output responses were measured with increasing stimulus intensity from 10 to 100 μA at a 10-μA interval. PPF was measured with indicated interpulse interval ranging from 10 to 250 ms. The baseline was defined by the first 20-min recording under 0.017 Hz and 0.1-ms pulse duration, then LTP was evoked by 1 or 4 trains of 1-s 100-Hz high-frequency stimulation (1X HFS or 4X HFS) with 5-min inter-train intervals. For field-recording on hippocampal slices prepared from AAV-injected brains, GFP signal in the stratum lacunosum moleculare or the CA1 cell layer was used to confirm the delivery of AAV at the EC or CA1 region, respectively. Only GFP-positive slices were used for electro-recording.

### Primary neuron culture and luciferase reporter assay

Cortical and hippocampal tissues of E18.5 embryos from *Cpeb2*^+/-^ heterozygous mating or cortical tissues of E18.5 rat embryos were collected for neuron culture as reported [35]. The percent of neurons in DIV14-DIV22 cultures was more than 85% based on counting the number of MAP2-positive neurons and GFAP-positive glia. Neurons were seeded at 3 × 10^6^ cells/60-mm dish for immunoblotting and 10^6^ cells/35-mm dish for RNA isolation by using TRIzol. Neurons were plated at 10^5^ or 5 × 10^5^ cells/18-mm coverslip in a 12-well plate for fluorescence in situ hybridization or FM4-64FX loading assay, respectively, and 7 × 10^5^ cells/well in a 12-well plate for 4X HFS-induced protein expression. The *Slc17a6* 3'-UTR was amplified from mouse brain cDNA with the primers 5'-GGACTAGTCCGATGCTAGTTGCTGGATTCATTTG and 5'-CGGGATCCGTTTTGATTTTACAAAAGGGTAAATAGAA, and cloned into the pGL3-promoter vector (Promega). Human embryonic kidney 293 T (HEK293T) or Neuro-2a (N2a) cells were transfected with 0.5 μg plasmid mixture for dual luciferase assay (Promega).

### Immunohistochemistry, imaging acquisition, and quantification

The detailed procedures were previously described [26, 35]. Briefly, brains isolated from mice after cardiac perfusion of PBS and 4% formaldehyde/PBS were post-fixed in 4% formaldehyde overnight, then immersed in 30% (w/v) sucrose/PBS at 4 °C overnight. Brains were embedded in Tissue-Tek OCT compound and sectioned coronally at 30 μm or 10 μm by using a Leica cryostat. Selected sections were processed for immunohistochemistry (30-μm thickness) with indicated primary antibodies (Additional File 1: Table S1) or FISH (10-μm thickness) as described in the next paragraph. Images were acquired by AxioImager Z1 (Zeiss) or a laser-scanning LSM780 (Zeiss) confocal microscope and quantified by using ImageJ (NIH).

### Fluorescence in situ hybridization (FISH)

Stellaris FISH Probes, mouse *Slc17a6* with CAL Fluor Red 610 (VSMF-30156–5, LGC Biosearch Technologies), were hybridized to brain slices or cultured neurons, following the manufacturer’s instructions and the published study with modifications [49]. In brief, all solutions were prepared in diethyl pyrocarbonate (DEPC)-based PBS unless otherwise specified. DIV17 neurons on coverslips were fixed with 2% formaldehyde for 15 min, then washed with PBS for 5 min × 3 times. Fixed samples were permeabilized with 0.3% Triton X-100 for 10 min, washed with PBS for 3 times and then rinsed 3 times with the hybridization buffer (30 mM sodium citrate pH 7, 0.3 M NaCl, 10% dextran sulfate, 2 mM ribonucleoside vanadyl complex, 10% formamide, 200 μg/ml tRNA, 80 μg/ml bovine serum albumin [BSA] in DEPC-H_2_O). The FISH Probes were diluted to a final 0.25 μM in the blocking buffer (1% w/v Blocking Reagent [11096176001, Roche], 10 mM maleic acid pH 7.5, 15 mM NaCl, 0.2 mM Tris–HCl pH 8, 20 μM EDTA in hybridization buffer) along with the designated primary antibodies (Additional File 1: Table S1). After 37 °C hybridization in a humidified dark chamber for at least 16 h, samples were washed with 0.3% Triton X-100 in PBS for 5 min × 3 times, followed by incubation with secondary antibodies for 1 h. After one rinse with PBS, samples were post-fixed with 2% formaldehyde for 15 min and washed with PBS for 5 min × 3 times before mounting with ProLong Gold Antifade Mountant (ThermoFisher). Similar procedures were performed to detect *Slc17a6* RNA in brain sections, with the inclusion of 1 μg/ml proteinase K treatment at 37 °C for 10 min subsequent to permeabilization in 0.3% Triton X-100 to enhance RNA detection.

### Activity-induced FM4-64FX loading assay

DIV17-23 neurons on coverslips or in microfluidic chambers were activated ± 4X HFS ± 200 μg/ml cycloheximide, then harvested at designated times for immunoblotting or incubated for 2 h, followed by the loading of FM4-64FX as described [50]. All solutions were prepared in Tyrode’s buffer (25 mM HEPES pH 7.4, 119 mM NaCl, 2.5 mM KCl, 2 mM CaCl_2_, 2 mM MgCl_2_ and 30 mM D-glucose) unless otherwise indicated. Briefly, coverslips were rinsed twice with Tyrode’s buffer and once with the loading buffer (10 μM FM4-64FX [Thermo Fisher], 50 μM APV and 10 μM CNQX) and then stimulated for 90 s of 10-Hz in the same buffer. After 20-min incubation, neurons were washed twice with Tyrode’s buffer, then incubated for 10 min in 1 mM ADVASEP-7 (binds and removes dye from external membranes, Merck), 50 μM APV and 10 μM CNQX [51]. After one wash of Tyrode’s buffer, neurons were fixed with 4% formaldehyde/PBS for 10 min, permeabilized with 0.2% Triton X-100/PBS for 10 min, and blocked with 5% BSA/PBS for 1 h, followed by overnight incubation of primary antibodies (Additional File 1: Table S1) in 1% BSA/PBS at 4 °C, then secondary antibodies for 2 h at room temperature before mounting with ProLong Gold Antifade Mountant.

### Synaptosome preparation and stimulation

All steps were performed at 4 °C unless otherwise specified, and buffers were oxygenated with 95% O_2_ and 5% CO_2_ for approximately 2 h before use. Cortical and hippocampal tissues of adult mice were fractionated to obtain crude synaptosomes [26]. Tissues were homogenized (600 μl buffer/100 mg tissue) in the buffer containing 8.33 mM D-glucose, 250 mM sucrose, 20 mM HEPES pH 7.4, 25 mM Tris–HCl pH 7.4, 10 mM KCl, 1.5 mM MgCl_2_, 1 mM EDTA, 1 mM DTT, 1X protease inhibitor (Sigma-Aldrich), 1X PhosSTOP (Roche), and 60 U/ml RNase Inhibitor (TOOLS), by using a Dounce homogenizer with the loose pestle for 50 strokes twice at an interval of 5 min. The homogenates were centrifuged at 300 *xg* for 2 min to remove debris, 700 *xg* for 5 min to remove nuclei, and then 9200 *xg* for 20 min to collect pellets as crude synaptosomes, which were resuspended in Krebs buffer (212.7 mM D-glucose, 118.5 mM NaCl, 24.9 mM NaHCO_3_, 1.18 mM Na_2_SO_4_, 1.18 mM KH_2_PO_4_, 2.5 mM CaCl_2_, 15 μl/ml 1N HCl, 1X protease inhibitor, 1X PhosSTOP, and 60 U/ml RNase Inhibitor) and aliquoted into 200 μl per tube. Synaptosomal samples were pre-warmed at 37 °C for 5 min, then stimulated without (Ctrl) or with 50 μM NMDA and 10 μM glutamate for 30 s, followed by the addition of 120 μM APV ± 200 μg/ml cycloheximide, then incubated at 37 °C for designated times according to the published protocol [52]. All samples were centrifuged at 9200 *xg* for 20 min to collect synaptosomal pellets, which were lysed and sonicated in the buffer containing 50 mM Tris–HCl pH 6.8, 0.2% Triton X-100, 0.04% sodium deoxycholate, 1.5% SDS and 5% glycerol, then used for immunoblotting.

### Immunoblotting and quantification

Adult mouse cortex or DIV21 cultured neurons was homogenized in the lysis buffer (50 mM HEPES pH 7.4, 150 mM NaCl, 1 mM MgCl_2_, 0.5% Triton X-100, 1 mM DTT, 1 mM EDTA, 1 mM EGTA, 2X protease inhibitor [Sigma-Aldrich] and 1X PhosSTOP [Roche]) for 30 min on ice before centrifugation at 16,000 *xg* for 5 min at 4 °C. The protein concentration of cortical, neuronal and synaptosomal lysates was quantified by using the Bradford protein assay (Bio-Rad) or Pierce BCA assay kit (Thermo Fisher). Equal amounts of samples were mixed with Laemmli sample buffer and heated at 55 °C for 20 min, separated on 10% SDS-PAGE, then transferred to nitrocellulose membranes (Millipore). Primary antibodies were incubated overnight at 4 °C and then horseradish peroxidase-conjugated secondary antibodies (Additional File 1: Table S1). Immunostained signals detected with chemiluminescence substrates (Millipore) were acquired by using ImageQuant LAS 4000 Mini (GE Healthcare) and then quantified by using ImageJ (NIH).

### RNA immunoprecipitation (RIP) and quantitative RT-PCR (RT-qPCR)

The previous protocols were followed with modifications [30, 35]. In brief, cortical and hippocampal lysates from adult male mice were divided and incubated with control or CPEB2 IgG-bound protein G beads for 3 h. One tenth of the beads were kept for immunoblotting, and the rest were isolated for RNA for microarray analysis. Similarly, to validate selected CPEB2-binding targets, hippocampal lysates from adult male mice were used. One third of the beads were used for immunoblotting, and the rest were isolated for RNA for RT-qPCR. Total RNA from adult cortex and DIV21 neurons were isolated by TRIzol (Invitrogen). RT-qPCR was performed by using ImPromII Reverse Transcriptase (Promega) and Universal Probe Library and Lightcycler 480 system (Roche). Primers and probes for qPCR are: *Slc17a6*, 5'-TGTTGGTGCAATGACAAAGAA, 5'-CCTGAGGCAAATAGTGCATAAA, and UPL probe #22; *Slc17a7*, 5'-GCAGGAGGAGTTTCGGAAG, 5'-CCTGCCGCTTCTCCAGTA, and UPL probe #75; *Syt1*, 5'-GCGATCTCCAGAGTGCTGAG, 5'-GACAACAGTCAGCTTGCCG, and UPL probe #7; *Gapdh*, 5'-GCCAAAAGGGTCATCATCTC, 5'-CACACCCATCACAAACATGG, and UPL probe #29.

### RIP-microarray and gene ontology (GO) analysis

The procedure with use of RIP samples for microarray analysis (Agilent SurePrint G3 Mouse GE 8 × 60 K, Welgene, Taiwan) was described before [30]. Briefly, 2,685 transcripts with raw signals > 1000, > 1.5-fold enrichment in CPEB2 IgG-precipitated substances, and at least one CPE in 3'-UTR were identified from 2 independent RIP-microarray experiments (Additional File 2). The learning-associated translatome (GSE124592) was reanalyzed to identify 2,438 genes, showing ± 1.5-fold change in expression only in the axonal compartment after fear learning (Additional File 3). CPEB2-bound axon-specific transcripts were identified by cross-comparison of 2,685 CPEB2-RIP targets with 2,438 axon-translated mRNAs (Additional File 4) and then GO-analyzed by Metascape [53] with the key word ‘glutamatergic transmission’ included to filter the results (Additional File 4).

### Statistics

Analysis was performed using SigmaPlot software. The statistical methods and analyzed results are listed (Additional File 5).

## Results

### CPEB2 regulated both pre- and post-synaptic transmission in the SC pathway

**Fig. 1:**
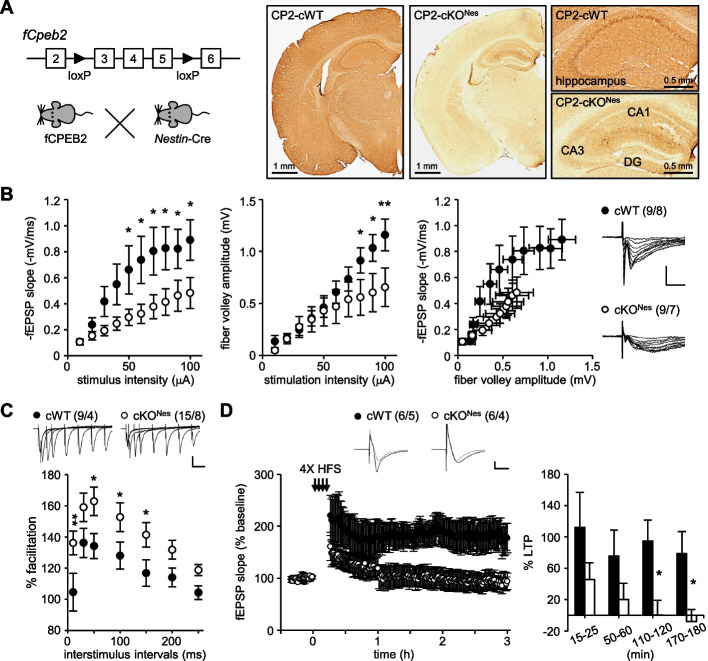
Aberrant synaptic transmission and LTP in CPEB2-cKO^Nes^ SC-CA1 pathway. **A** Mice carrying the *Cpeb2* allele with two loxP sites flanking exons 3–5 (fCPEB2) were crossed with *Nestin*-Cre mice to generate CPEB2-cWT and -cKO^Nes^ mice. CPEB2 immunohistochemistry of coronal brain slices was from adult CPEB2-cWT and -cKO^Nes^ mice. **B**-**D** Electrophysiological responses in the SC-CA1 pathway of adult (3- to 5-month-old) mice, including the input–output curve of the fEPSP slope and fiber volley amplitude in response to increasing stimulus intensity (**B**), paired-pulse facilitation (**C**), and LTP evoked by 4X HFS (**D**). Sample traces were the average fEPSP of 5 min before (gray) and the very last 5 min (black) after stimulation. Numbers in parentheses (n/N) represent the number of recorded slices (n) and mice (N). Sample traces with vertical scales of 0.5 mV and horizontal scales of 10 ms (i-o curve and LTP) and 50 ms (PPF). Data are mean ± SEM. *P < 0.05 and **P < 0.01, two-way ANOVA with Fisher’s LSD *post-hoc* comparison

To explore an RBP-mediated presynaptic/axonal translation for plasticity and memory, we first examined whether CPEB2 affects presynaptic electrophysiological responses in hippocampal slices. We used a *Nestin*-Cre mouse line to generate CPEB2-cKO^Nes^ mice with *Cpeb2* ablated in most neurons and glia including those in CA1 and CA3 regions (Fig. 1A). In our previous study, CPEB2 was only depleted in hippocampal CA1 pyramidal neurons but not CA3 in CPEB2-cKO^Camk2^ mice. Hence, they only exhibited impaired postsynaptic responses and long-lasting LTP due to lack of postsynaptic CPEB2 in the SC pathway [35]. Here, we measured the input–output relationship in the SC pathway which measures presynaptic (fiber volley [FV] amplitude) and postsynaptic (the slope of field excitatory postsynaptic potential [fEPSP]) responses of CA3 and CA1 neurons, respectively. Both fEPSP slopes (F_(1,160)_ = 32.264, *P* < 0.001) and FV amplitudes (F_(1,160)_ = 9.928, *P* = 0.002) were reduced in CPEB2-cKO^Nes^ mouse hippocampal slices (Fig. 1B). Moreover, PPF, which monitored presynaptic neurotransmitter release and was inversely correlated with the release probability [54], was enhanced in CPEB2-cKO^Nes^ mouse hippocampal slices (F_(1,154)_ = 27.033, *P* < 0.001, Fig. 1C). Translation-dependent LTP elicited by 4X HFS [55] lasted for 3 h in the CPEB2-cWT but not CPEB2-cKO^Nes^ SC pathway (F_(1,40)_ = 15.149, *P* < 0.001, Fig. 1D). As compared with what we previously observed in CPEB2-cKO^Camk2^ mice [35], the additional deletion of CPEB2 in CA3 neurons of CPEB2-cKO^Nes^ mice caused abnormal presynaptic responses, including FV amplitude and PPF (Fig. 1B, C), and failed to maintain long-lasting LTP for more than 1 h (Fig. 1D), rather than merely decreasing LTP magnitude in CPEB2-cKO^Camk2^ mice [35], so CPEB2 also played a presynaptic role in modulating glutamatergic transmission.

### CPEB2-cKO^Nes^ mice exhibited defective hippocampus-dependent learning and memory

To assess whether CPEB2-cKO^Nes^ mice exhibited cognitive impairment, we conducted behavioral assays previously used in CPEB2-cKO^Camk2^ mice, which showed defective consolidation of spatial memory in the Morris water maze test and fear memory after contextual fear conditioning [35], but excluded the fear conditioning test because hemizygous *Nestin*-Cre mice showed defective contextual- and cued-conditioned fear [45]. The exploratory activity in the open field (Additional File 1: Fig. S1A) and the anxiety level in the elevated plus maze (Additional File 1: Fig. S1B) were comparable between CPEB2-cKO^Nes^ mice and their cWT littermates. After 4-day acquisition training in the Morris water maze, both groups learned to find the hidden platform in the target quadrant (F_(3,52)_ = 14.609, *P* < 0.001), but CPEB2-cKO^Nes^ mice performed worse than their littermates (F_(1,52)_ = 7.173, *P* = 0.010, Additional File 1: Fig. S1C). In the day 5 probe test, we analyzed the percentage of time mice spent in the target quadrant where the platform was previously placed. CPEB2-cKO^Nes^ mice showed reduced memory consolidation (F_(3,52)_ = 4.182, *P* = 0.010, *P* = 0.020 in target quadrant, Additional File 1: Fig. S1D), but their swimming ability and visual acuity were normal (Additional File 1: Fig. S1E).

### Systemic identification of CPEB2-bound transcripts involved in learning-associated presynaptic translation

**Fig. 2:**
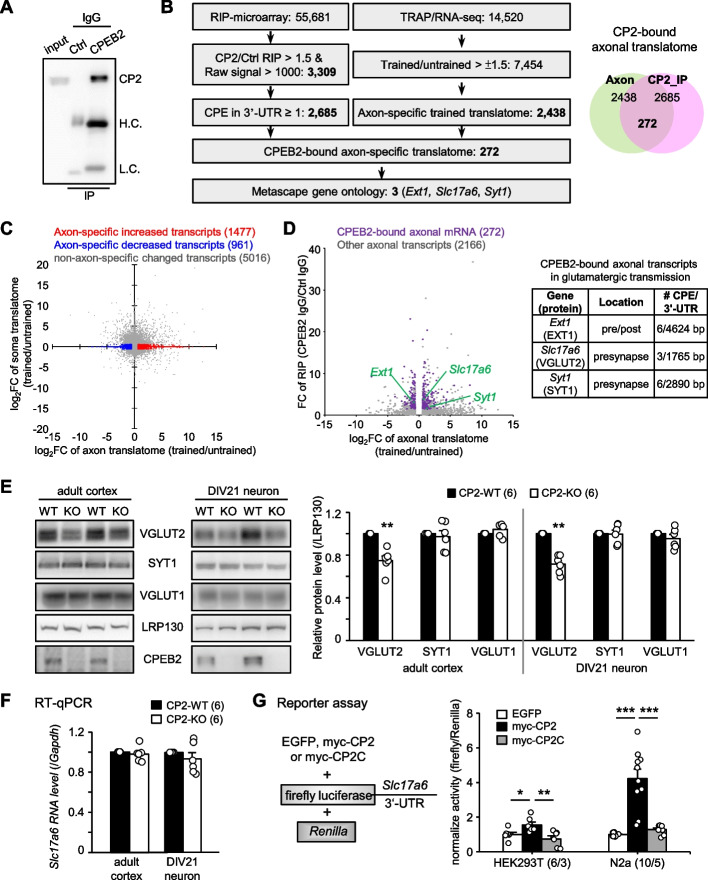
Identification and validation of learning-associated presynaptic *Slc17a6* is translationally upregulated by CPEB2. **A** Adult mouse cortices and hippocampi were used for RNA immunoprecipitation with CPEB2 or control (Ctrl) IgG. The precipitated substances were processed for western blot analysis or RNA isolation for microarray assay. **B** The flowchart denoted the criteria used to compare the CPEB2-immunoprecipitated transcriptome (GSE208738) and the learning-related translatome (GSE124592). **C** Among 7454 transcripts, 2438 showed more than 1.5-fold change in expression only in the axonal compartment: 1477 were upregulated (red dot) and 961 downregulated (blue dot). **D** Among the 2438 axon-specific translated mRNAs, 272 were CPEB2-binding targets with at least one CPE in their 3'-UTRs (purple and green dots). Three were known to function at glutamatergic presynapses and contain multiple CPEs in their 3'-UTRs of varying lengths. **E** Adult cortices and cultured neurons at 21 days in vitro (DIV) from global CPEB2-wild type (CPEB2-WT) and -KO mice were used for immunoblotting. The protein levels were normalized to that of LRP130. **F** RT-qPCR analysis of *Slc17a6* mRNA level (normalized to *Gapdh* level) in adult cortex and DIV 21 neurons. **G** Dual luciferase reporter assay. The firefly luciferase reporter appended with mouse *Slc17a6* 3'-UTR and *Renilla* luciferase plasmids were co-transfected with the plasmid expressing EGFP, myc-tagged full-length (CP2) or C-terminus (CP2C) of CPEB2 into HEK293T or N2a cells (duplicate transfections in 3 or 5 independent cultures, respectively). Data are mean ± SEM. *P < 0.05, **P < 0.01 and ***P < 0.01, one-way ANOVA with Fisher’s LSD *post-hoc* test or Student’s* t* test

Since SC axons were transected from the soma of CA3 neurons during electro-recording and additional depletion of presynaptic CPEB2 severely impaired protein synthesis-dependent LTP (Fig. 1D), CPEB2 must regulate axonal translation to support enduring neurotransmitter release for LTP. To identify the candidate mRNAs regulated by CPEB2, we used RIP with adult mouse cortical and hippocampal tissues. CPEB2- and control IgG-precipitated materials were used for immunoblotting of CPEB2 (Fig. 2A) and RNA isolation for microarray analysis. From 2 independent RIP experiments, 3,309 transcripts with raw signal > 1,000 were enriched more than 1.5-fold in CPEB2-immunoprecipitated samples, and 2,685 contained at least 1 CPE in their 3'-untreanslated region (3'-UTR) (Fig. 2B and Additional File 2). To investigate whether CPEB2 could regulate the translation of specific axonal mRNAs to affect presynaptic plasticity and memory, we cross-compared CPEB2-bound transcriptome with the only learning-associated axonal translatome (GSE124592). In this study, Ostroff et al. used the TRAP method [56] to isolate ribosome-associated transcripts (i.e., translatome) in axons and somas of auditory cortical TE3 neurons before and after cued fear conditioning [20]. We analyzed 14,520 TRAP-isolated transcripts from this dataset and found 7,454 transcripts with more than ± 1.5-fold change after fear conditioning (Fig. 2B and Additional File 3). Moreover, a total of 2,438 transcripts in the trained translatome showed changed expression only in the axonal compartment (Fig. 2C and Additional File 3). Cross-comparison of CPEB2-bound transcriptome with the 2,438 transcripts identified 272 mRNAs being CPEB2-bound and axon-translated targets (Fig. 2D and Additional File 4). Because of aberrant glutamatergic transmission in the CPEB2-cKO^Nes^ SC circuit (Fig. 1), we included the ‘glutamatergic transmission’ keyword in Metascape gene annotation analysis [53] and identified 7 candidates (Additional File 4). We excluded 4 of them, with expression mainly in astrocytes (i.e., *Glul*) [57] or whose function was mainly at the postsynapse (i.e., *Homer1*, *Gria3* and *Grin3a*) [58–61]. The remaining 3 candidates, *Ext1, Slc17a6* and *Syt1*, encode proteins localized in the presynaptic compartment and contain 6, 3 and 6 CPEs, respectively, in their 3'-UTRs (Fig. 2D). *Ext1* encodes exostosin glycosyltransferase 1, which functions in both pre- and post-synaptic domains [62, 63], so we focused on *Slc17a6* and *Syt1*, which encode VGLUT2 for transporting glutamate to synaptic vesicles [64, 65] and SYT1 for calcium-dependent exocytosis of glutamate- or GABA-containing vesicles [66–68].

### Expression of VGLUT2 was translationally activated by CPEB2

VGLUT2 and SYT1 are exclusively concentrated in synaptic vesicles, so they may rely on local translation to maintain their presynaptic demands. Using CPEB2-WT and -KO adult cortices and cultured neurons, we identified decreased protein level of VGLUT2 but not SYT1 (Fig. 2E) and the change was at the protein rather than mRNA level (Fig. 2F). VGLUT1, encoded by *Slc17a7*, is more widely expressed than VGLUT2 in the hippocampus and cortex (Additional File 1: Fig. S2A) and also takes up glutamate into synaptic vesicles [64, 65]. Although the 3'-UTR of VGLUT1 mRNA contained no CPE and was not enriched in the CPEB2-immunoprecipitated transcriptome, we also examined and found that CPEB2 deficiency did not affect VGLUT1 level (Fig. 2E). Moreover, using the firefly luciferase reporter appended to the 3'-UTR of mouse *Slc17a6*, we found that the full-length but not C-terminal RNA-binding domain of CPEB2 enhanced the synthesis of firefly luciferase as compared with the GFP control (Fig. 2G). Therefore, CPEB2 bound to and promoted the translation of VGLUT2 mRNA.

### CPEB2 regulated synaptic plasticity in the TA-CA1 pathway

**Fig. 3:**
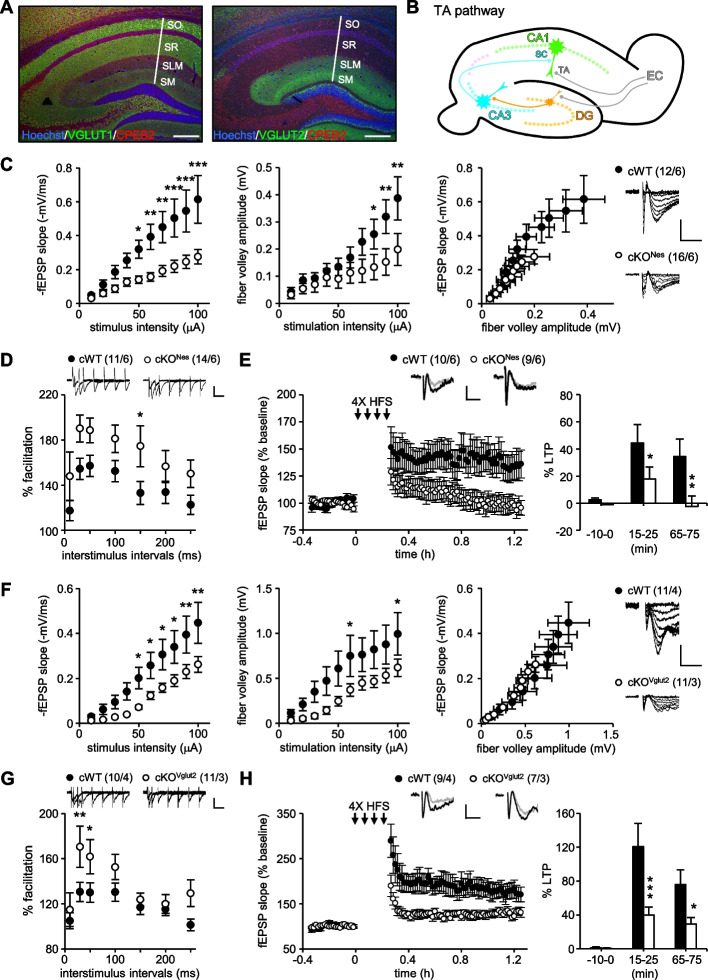
CPEB2 deficiency affects glutamatergic transmission and translation-dependent LTP in the TA-CA1 circuit. **A** Differential distribution of VGLUT1^+^ and VGLUT2^+^ afferents on hippocampal CA1 neurons. SO, stratum oriens; SR, stratum radiatum; SLM, stratum lacunosum moleculare; SM, stratum moleculare. Scales, 0.2 mm. **B** The illustration denoted SC-CA1 and TA-CA1 pathways. **C**-**H** CPEB2-cKO^Nes^ and -cKO^Vglut2^ male mice (3 to 5 months old) were used for field recording. The input–output responses (**C** and **F**), paired-pulse facilitation (**D** and **G**) and 4X HFS-evoked LTP (**E** and **H**) in the TA-CA1 pathway of CPEB2-cKO^Nes^ and -cKO^Vglut2^ hippocampal slices, respectively. Numbers in parentheses (n/N) represent the number of recorded slices (n) and mice (N). Sample traces were presented in the same manner as described in Fig. 1. Data are mean ± SEM. *P < 0.05, **P < 0.01 and ***P < 0.001, two-way ANOVA with Fisher’s LSD *post hoc* test

Hippocampal CA1 neurons receive glutamatergic afferents from CA3 neurons in the stratum radiatum and from layer III entorhinal cortex neurons in the stratum lacunosum moleculare [69]. Because most presynaptic terminals express VGLUT2 in the stratum lacunosum moleculare (Fig. 3A and Additional File 1: Fig. S2A), we then determined whether CPEB2-controlled protein synthesis, such as for VGLUT2, was important for presynaptic glutamate release and plasticity by recording the TA-CA1 circuit [47, 48] (Fig. 3B). Similar to what we observed in the SC circuit (Fig. 1), the input–output curve of CPEB2-cKO^Nes^ slices was diminished in both fEPSP slopes (F_(1,260)_ = 58.060, *P* < 0.001) and FV amplitudes (F_(1,260)_ = 17.149, *P* < 0.001, Fig. 3C) and the CPEB2-cKO^Nes^ TA-CA1 pathway exhibited enhanced PPF (F_(1,161)_ = 19.319, *P* < 0.001, Fig. 3D) and defective 4X HFS-evoked LTP (F_(1,51)_ = 8.771, *P* = 0.005, Fig. 3E). We then used a *Vglut2*-Cre mouse line to generate CPEB2-cKO^Vglut2^ mice and investigated TA-CA1 plasticity under CPEB2 deficiency in the subset of glutamatergic neurons. Due to transient expression of VGLUT2 in the developing hippocampus [64, 65, 70], CPEB2 was depleted in most pre- (i.e., entorhinal cortex) and post-synaptic (i.e., hippocampus) glutamatergic neurons in the TA-CA1 pathway (Additional File 1: Fig. S2B), including all CaMK2α-expressing CA1 neurons (Additional File 1: Fig. S2C). Similarly, we observed diminished fEPSP slopes (F_(1,200)_ = 35.106, *P* < 0.001) and FV amplitudes (F_(1,200)_ = 25.076, *P* < 0.001, Fig. 3F), enhanced PPF (F_(1,133)_ = 12.826, *P* < 0.001, Fig. 3G) and defective 4X HFS-evoked LTP (F_(1,42)_ = 11.022, *P* = 0.002, Fig. 3H) in CPEB2-cKO^Vglut2^ TA-CA1 circuit. However, protein synthesis-independent LTP elicited by 1X HFS was unaffected in CPEB2-cKO^Nes^ and -cKO^Vglut2^ slices (Additional File 1: Fig. S3A and S3B), reinforcing the role of CPEB2 in translation-dependent long-term synaptic plasticity [35].

### CPEB2-cKO^Vglut2^ mice showed defective memory consolidation

**Fig. 4:**
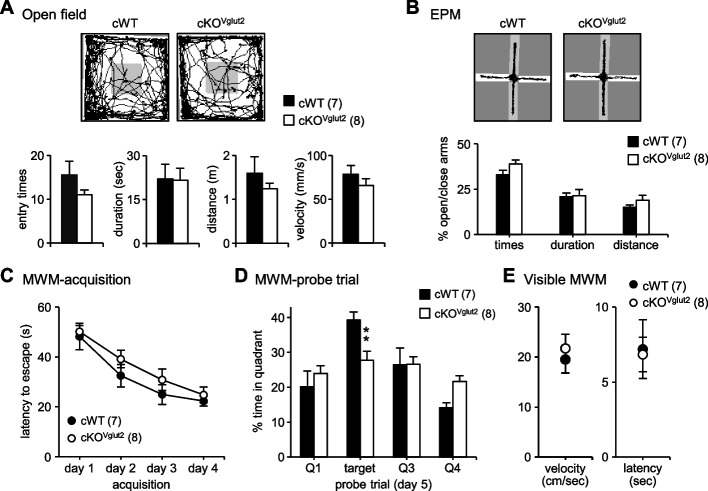
Impaired spatial memory consolidation in CPEB2-cKO^Vglut2^ mice. **A**-**E** Adult male mice at 2 to 4 months old were used for behavior assays. **A** Open-field. Representative moving traces in the open arena during the first 10 min and the quantified entry times, duration, moving distance and velocity. **B** Elevated plus maze (EPM). Representative 5-min moving traces in the EPM and the quantified entry times, duration and moving distance in the open versus closed arms. **C** Morris water maze (MWM). Mice were trained with 4 trials per day for 4 consecutive days, and the escape latency to the platform was averaged from 4 trials. **D** The MWM probe test on day 5 recorded the percentage of time (total 60 s) spent in each quadrant. **E** In the visual MWM task, the velocity and latency reflected the swimming ability and visual acuity, respectively. Data are mean ± SEM. **P < 0.01, Student’s *t* test and two-way ANOVA with Fisher’s LSD *post-hoc* test. The number of mice for behavioral assays was denoted in parentheses

The loss of CPEB2 in glutamatergic neurons sufficiently impaired glutamatergic transmission and long-term plasticity (Fig. 3F-H), so we examined the behavior of CPEB2-cKO^Vglut2^ mice and found no abnormalities in their exploratory activity in the open field (Fig. 4A), anxiety level in the elevated plus maze (Fig. 4B), and spatial learning in the Morris water maze (Fig. 4C). However, CPEB2-cKO^Vglut2^ mice had reduced memory consolidation (F_(3,52)_ = 4.127, *P* = 0.011, *P* = 0.006 in target quadrant, Fig. 4D) despite normal swimming ability and visual acuity (Fig. 4E).

### CPEB2 regulated both pre- and post-synaptic plasticity in the TA-CA1 pathway

**Fig. 5:**
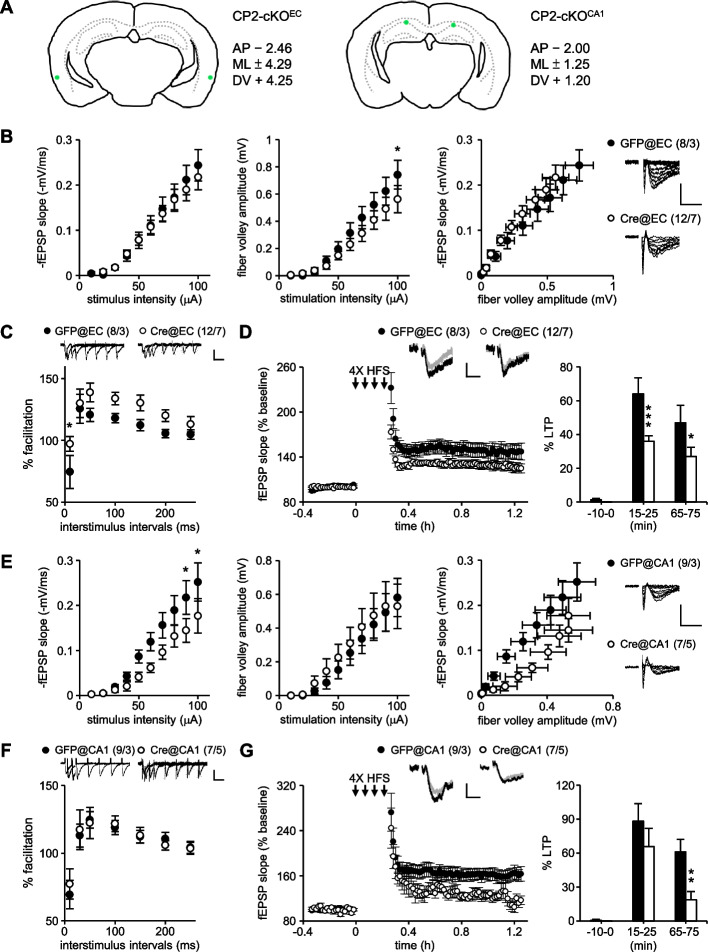
Pre- or post-synaptic deletion of CPEB2 impairs glutamatergic transmission and LTP in the TA-CA1 circuit. **A** The virus injection sites in the entorhinal cortex (EC, left) and CA1 (right) were marked in green dots. **B**-**G** CPEB2-cWT mice were injected with AAV8-GFP (4.2 × 10^9^ vg) or AAV9-Cre-ires-GFP (1.62 × 10^10^ vg) to generate cWT control or CPEB2-cKO^EC^ and -cKO^CA1^ mice, respectively. Four weeks after intracranial delivery of AAV, 3–5-month-old male mice were used for field recording. The input–output responses (**B** and **E**), paired-pulse facilitation (**C** and **F**) and 4X HFS-evoked LTP (**D** and **G**) in the TA-CA1 pathway of CPEB2-cKO^EC^ and -cKO^CA1^ hippocampal slices, respectively. Numbers in parentheses (n/N) represent the number of recorded slices (n) and mice (N). Sample traces were presented in the same manner as described in Fig. 1. Data are mean ± SEM. *P < 0.05, **P < 0.01 and ***P < 0.001, two-way ANOVA with Fisher’s LSD *post hoc* test

Because CPEB2-activated GRASP1 mRNA translation is important for recycling of AMPARs to postsynaptic sites of CA1 neurons [35], impaired translation-dependent LTP in the SC (Fig. 1D) and TA circuits (Fig. 3E, H) of CPEB2-cKO^Nes^ and -cKO^Vglut2^ hippocampi could be attributed to both pre- and post-synaptic dysregulation. To determine whether the loss of CPEB2 at the presynaptic TA afferents (CPEB2-cKO^EC^) or the postsynaptic CA1 neurons (CPEB2-cKO^CA1^) sufficiently affected translation-dependent LTP, we injected adeno-associated virus expressing GFP or Cre recombinase-ires-GFP (Cre) at the lateral entorhinal cortex (@EC) or the CA1 region (@CA1) of CPEB2-cWT mice, respectively. The injection sites were revealed by the expression of GFP (Fig. 5A) and recordings were conducted using only slices of GFP signal in TA afferents onto the stratum lacunosum moleculare (@EC) or in CA1 neurons (@CA1). CPEB2 deficiency in TA afferents (Cre@EC) reduced FV amplitudes (F_(1,180)_ = 6.095, *P* = 0.014, Fig. 5B), enhanced PPF (F_(1,126)_ = 12.933, *P* < 0.001, Fig. 5C) and decreased 4X HFS-evoked LTP (F_(1,54)_ = 12.656, *P* < 0.001, Fig. 5D) but did not affect 1X HFS-induced LTP (Additional File 1: Fig. S3C). On the other hand, depleting CPEB2 in postsynaptic CA1 (Cre@CA1) reduced fEPSP slopes (F_(1,140)_ = 15.193, *P* < 0.001, Fig. 5E) and 4X HFS-evoked LTP (F_(1,42)_ = 6.289, *P* = 0.016, Fig. 5G). To verify that the effect of AAV-Cre on reducing 4X HFS-elicited LTP in CPEB2-cWT mice was not caused by Cre-induced toxicity or by using different AAV serotypes to express GFP or Cre-ires-GFP, we performed experiments in wild-type mice with AAV-injected @EC. No differences were found in 4X HFS-evoked LTP regardless of whether AAV9-GFP (1.62 × 10^10^ vg), AAV9-Cre-ires-GFP (1.62 × 10^10^ vg) or AAV8-GFP (0.42 × 10^9^ vg) was used (Additional File 1: Fig. S4). Additionally, cWT mice but not WT mice infected with AAV9-Cre-ires-GFP @EC, exhibited reduced 4X HFS-induced LTP in the TA-CA1 pathway (Additional File 1: Fig. S4D). These results supported the critical role of CPEB2-controlled translation in both pre- and post-synaptic compartments to regulate glutamatergic plasticity.

### Activity-triggered synaptosomal VGLUT2 synthesis depended on CPEB2

**Fig. 6:**
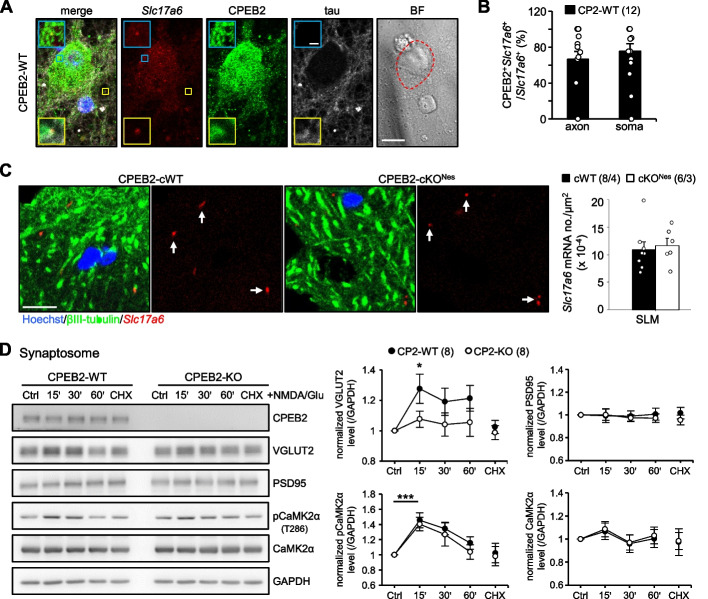
CPEB2 is present in most *Slc17a6* mRNA puncta and promotes VGLUT2 synthesis in NMDA/glutamate-stimulated synaptosomes. **A** DIV17 neurons were probed with *Slc17a6* mRNA by FISH, followed by immunostaining of CPEB2 and the axonal marker tau and Hoechst labeling. Representative images showed the colocalization of *Slc17a6* mRNA and CPEB2 in somas (defined by bright-field images) and axons (tau^+^ areas) with magnified images outlined in blue and yellow squares, respectively. Scales, 10 μm and 1 μm in magnified images. **B** The percentage of *Slc17a6*^+^ puncta containing CPEB2 signal in neurons. Twelve image fields from 4 independent cultures were quantified. **C** Representative images and quantified results showed the number of *Slc17a6*^+^ puncta in the SLM layer of CPEB2-cWT and cKO^Nes^ hippocampi. Scale, 10 μm. Numbers in parentheses (n/N) represent the number of slices (n) and mice (N). **D** Synaptosomes from adult cortical and hippocampal tissues (8 independent preparations) were stimulated without (Ctrl) or with 50 μM NMDA and 10 μM glutamate for 30 s, followed by the addition of 120 μM APV ± 200 μg/ml cycloheximide (CHX) for the indicated times and then harvested for immunoblotting. Data are mean ± SEM. *P < 0.05 and ***P < 0.001, Mann–Whitney Rank Sum Test in (**B**), Student’s *t* test in (**C**), and two-way ANOVA with Fisher’s LSD *post-hoc* test in (**D**)

Aberrant presynaptic responses in severed CPEB2-lacking SC and TA afferents were similar to those reported in VGLUT2-cKO mice [70]. Because CPEB2 controls *Slc17a6* translation (Fig. 2E**-**G), we tested whether such a regulation could be axon-localized to affect the function of VGLUT2-containing vesicles. Therefore, we detected *Slc17a6* mRNA by FISH, followed by immunostaining of CPEB2 and an axonal marker, tau, in CPEB2-WT neurons (representative images in Fig. 6A). Because only a portion of pyramidal neurons express *Slc17a6* mRNA, we randomly selected 12 image fields with at least one soma expressing *Slc17a6* puncta for quantification. The number of *Slc17a6*-positive (*Slc17a6*^+^) puncta in somas and axons was counted and expressed as a relative percentage in each image field. Approximately 65% to 75% *Slc17a6*^+^ puncta contained a CPEB2 signal in both somatic and axonal compartments (Fig. 6B). Moreover, the number of *Slc17a6*^+^ puncta in the stratum lacunosum moleculare layer of CA1 neurons was comparable between CPEB2-cWT and -cKO^Nes^ hippocampi (Fig. 6C), indicating that CPEB2 deficiency did not affect axonal distribution of *Slc17a6* mRNA. Axonal colocalization of CPEB2 and *Slc17a6* mRNA supports the possibility of CPEB2-dependent local *Slc17a6* translation, so we isolated synaptosomes from cortical and hippocampal tissues and stimulated them with N-methyl-D-aspartate (NMDA) and glutamate to induce chemical LTP [52], which is accompanied by elevated phosphorylation of calcium/calmodulin-dependent protein kinase 2 subunit alpha (CaMK2α) at Thr 286 (Fig. 6D). CPEB2-WT but not CPEB2-KO synaptosomes showed NMDA/glutamate-induced expression of VGLUT2, and this increase could be blocked by cycloheximide, an inhibitor of eukaryotic translation (Fig. 6D).

### Reduced VGLUT2 in adult CPEB2-cKO^Nes^ and -cKO^Vglut2^ hippocampi

Since VGLUT2 was mainly detected in presynaptic axons of EC neurons projecting to hippocampal stratum lacunosum moleculare (Fig. 3A and Additional File 1: Fig. S2A), we used hippocampal tissues and confirmed that CPEB2 also bound to *Slc17a6* and *Syt1* transcripts but not *Slc17a7* mRNA (Additional File 1: Fig. S5A). Moreover, the protein level of VGLUT2 but not SYT1 and VGLUT1 was also diminished in CPEB2-cKO^Nes^ and -cKO^Vglut2^ adult hippocampi (Additional File 1: Fig. S5B). Therefore, CPEB2 binds to and enhances the expression of VGLUT2 in the TA afferents in the hippocampus.

### CPEB2 promoted axonal VGLUT2 synthesis to replenish VGLUT2^+^ vesicles

**Fig. 7:**
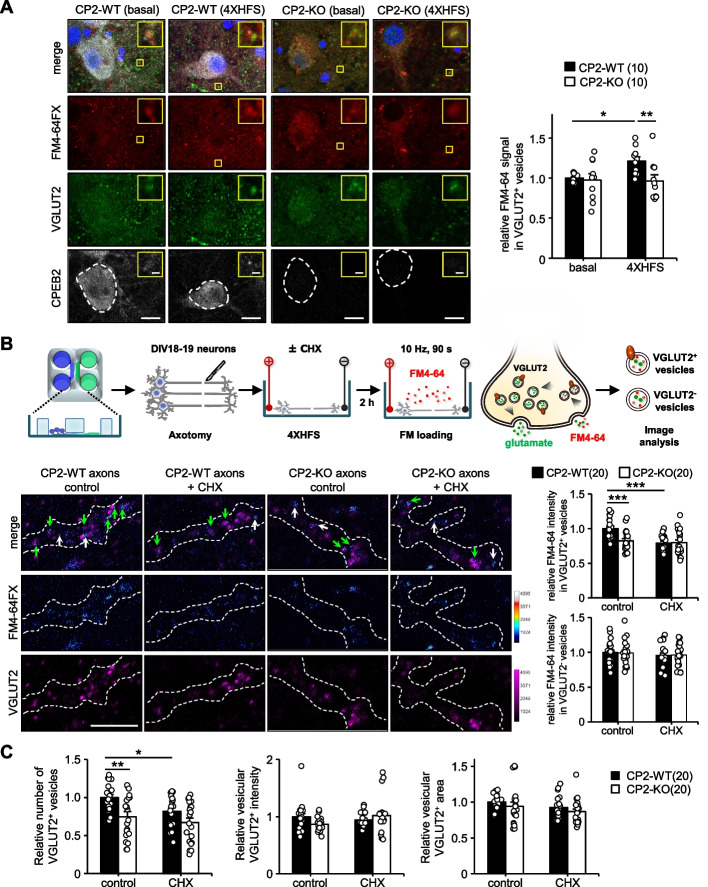
Activity- and CPEB2-dependent axonal VGLUT2 synthesis increases functional VGLUT2^+^ vesicles. **A** DIV17-23 neurons were stimulated without (basal) or with 4X HFS and incubated for 2 h before activity (10 Hz for 90 s)-stimulated FM4-64FX loading. The FM4-64FX puncta signals in VGLUT2^+^ vesicles were quantified in 10 image fields from 3 independent cultures and expressed as relative ratios. The region of interest at 4X magnification is shown in the insert. Scales, 10 μm and 1 μm in magnified images. **B**-**C** The experimental procedure was illustrated in the upper panel. CPEB2-WT and -KO neurons were seeded in microfluidic chambers and cultured for 18 to 19 DIV. After axotomy, the same stimulation and loading protocol were applied without (control) or with 50 μg/ml cycloheximide (CHX). The axonal region was outlined with an axonal marker, βIII-tubulin (marked by white dashed lines). The signal intensity of FM4-64FX and VGLUT2 was color-coded. The FM4-64FX puncta signals in VGLUT2^+^ and VGLUT2^−^ vesicles were quantified in 20 image fields from 5 independent cultures and expressed as a relative ratio. Data are mean ± SEM. *P < 0.05, **P < 0.01 and ***P < 0.001, two-way ANOVA with Fisher’s LSD *post-hoc* test. Scale, 5 μm

**Fig. 8:**
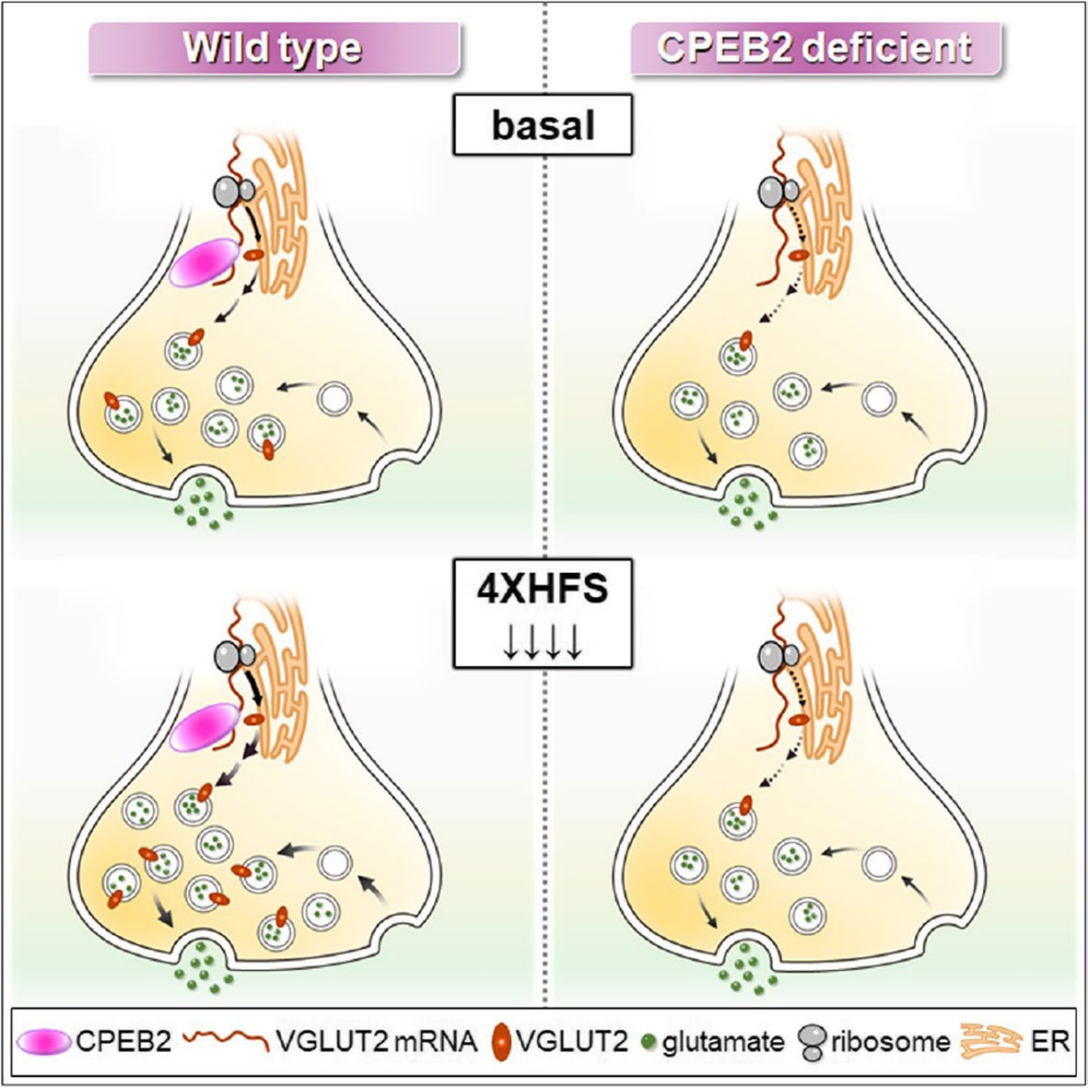
CPEB2-controlled axonal translation governs glutamatergic transmission and long-term presynaptic plasticity. CPEB2- and activity-dependent axonal translation of VGLUT2 mRNA replenishes synaptic vesicles and maintains presynaptic translation-dependent long-term potentiation in the TA-CA1 circuit

Defective protein synthesis-dependent LTP in the CPEB2-cKO^EC^ TA-CA1 pathway (Fig. 5D) strongly indicated that CPEB2-regulated presynaptic translation was important to sustain long-lasting LTP, in part, by affecting activity-induced expression of VGLUT2 in the transected TA axons (Fig. 6D). To support this notion further, we analyzed activity-elicited uptake of FM4-64FX dye into axonal VGLUT2^+^ vesicles in intact and axotomized neurons. We first confirmed that 4X HFS also increased VGLUT2 expression in CPEB2-WT but not CPEB2-KO cultured neurons (Additional File 1: Fig. S6), then analyzed the endocytosed FM4-64FX signal in VGLUT2^+^ vesicles after 900 pulse (10 Hz × 90 s)-evoked glutamate release. Therefore, only synaptic vesicles that undergo active release and recycling could be marked by the loading of FM4-64FX from the extracellular space. The loading of FM4-64FX into VGLUT2^+^ vesicles was comparable between CPEB2-WT and -KO neurons under the basal condition, but 4X HFS increased the FM4-64FX signal in VGLUT2^+^ vesicles only in CPEB2-WT neurons (Fig. 7A). To ensure that CPEB2-activated local *Slc17a6* translation is important to support the function of VGLUT2^+^ vesicles, neurons cultured in a microfluidic chamber [71], which only permits axons growing into the other side, were severed to separate axons from somas before 4X HFS (Additional File 1: Fig. S7A). Activity-enhanced FM4-64FX-loading signal in VGLUT2^+^ but not VGLUT2-negative (VGLUT2^−^) vesicles was sensitive to cycloheximide and defective in CPEB2-KO axons (Fig. 7B and Additional File 1: Fig. S7B). Moreover, the newly synthesized VGLUT2 molecules were mostly used to increase the pool of VGLUT2^+^ vesicles rather than increase VGLUT2 intensity or vesicular size of existing vesicles (Fig. 7C). Because VGLUT2 is a membrane protein, this finding agreed with the role of CPEB2 to promote translation of VGLUT2 mRNA presumably on ribosomes attached to endoplasmic reticulum and consequently produce new VGLUT2^+^ vesicles. Hence, activity- and CPEB2-dependent axonal translation was important to support the synaptic function of VGLUT2^+^ vesicles, thereby contributing to the persistence of long-term presynaptic plasticity (Fig. 8).

## Discussion

In this study, we revealed that CPEB2-controlled translation governs presynaptic plasticity and demonstrated that activity- and CPEB2-dependent axonal *Slc17a6* translation is critical to replenish VGLUT2 locally and support presynaptic glutamate transmission and translation-dependent LTP.

Unlike developing and regenerating axons, the axonal compartment of mature mammalian neurons is traditionally considered not to possess protein synthesis capacity [6]. However, because of its extensive architecture, activity-driven local synthesis of axonal proteins is intuitively appealing to support presynaptic plasticity in a timely and precise manner. By contrast to polysomes in dendritic spines, monosomes appear to dominate in the axonal compartment, and several omics studies with various approaches have demonstrated that the axonal translatome changes dynamically in response to activity input [10, 17–21]. The maintenance of several forms of memory-associated LTP requires de novo protein synthesis, including 4X HFS-evoked LTP in TA-CA1 and SC-CA1 pathways used in our study, but whether the newly produced proteins are contributed from presynaptic, postsynaptic or both compartments to support long-lasting synaptic strengthening is not entirely clear. Taking the example from our analysis, many learning-affected and axon-specifically translated mRNAs encode proteins in mitochondria to function in both pre- and post-synapses (Fig. 2C and Additional File 3), so identifying candidate mRNAs and determining which compartmental translation supports LTP is difficult. Moreover, most RBPs are widely expressed in the brain; region-specific ablation by crossing with different Cre transgenic mice or AAV delivery is needed to dissect this issue. For instance, the widely used *Camk2*-Cre mice [72] help in clarifying the postsynaptic contribution in the SC and TA circuits. In this study, we initially used AAV8-GFP as a control for AAV9-GFP-ires-Cre due to the strong neuronal tropism of both AAV serotypes after intracranial delivery [73]. However, several studies have indicated that AAV8 has higher glial tropism than AAV9 [73–76], so we conducted additional experiments (Additional File 1: Fig. S3C and Fig. S4D) to confirm that the defective LTP observed in Cre@EC mice (Fig. 5D) was not due to Cre toxicity or the differential tropisms of AAV8 and AAV9. Although no significant differences were observed between the use of AAV8-GFP and AAV9-GFP in our study, it is important to mention that using the same AAV serotype and titer is a standard practice in the field. Because presynaptic CPEB2 in VGLUT2-dominated TA circuit supported translation-dependent LTP (Fig. 5D), and 4X HFS- and CPEB2-driven axonal VGLUT2 synthesis increased the number of functional VGLUT2^+^ vesicles (Fig. 7B, C), so we expect that CPEB2-activated *Slc17a6* translation in transected TA axons is important to support long-lasting glutamate release to maintain LTP. Together with our previous finding in CPEB2-cKO^Camk2^ mice [35] and CPEB2-cKO^CA1^ (Fig. 5G), CPEB2 plays an important role in coordinating both pre- and post-synaptic translation for glutamatergic plasticity.

VGLUT2-KO mice die immediately after birth because of failure to generate respiratory rhythms [77, 78]. Similarly, most CPEB2-KO mice die postnatally because of elevated airway constriction induced by hyperparasympathetic signaling and impaired pulmonary development [40, 41]. We found no postnatal mortality in CPEB2-cKO^Vglut2^ mice, so the VGLUT2-glutamatergic circuit is unlikely defective in the respiratory system. By crossing with *Emx1*-Cre mice to ablate *Slc17a6* in excitatory neurons in the cortex and hippocampus, VGLUT2-cKO^Emx1^ mice showed impaired learning and memory in the Morris water maze and aberrant basal transmission, increased PPF and decreased LTP in the SC-CA1 circuit [70]. Despite many similarities in the SC-CA1 connection between VGLUT2-cKO^Emx1^ and CPEB2-KO^Nes^ mice, these electrophysiological defects are not merely caused by impaired VGLUT2 mRNA translation in CA3 neurons. Glutamatergic transmission in most excitatory synapses depends on VGLUT1 and VGLUT2, which display complementary expression in the adult brain (Fig. 3A and Additional File 1: Fig. S2A). However, in VGLUT2-cKO^Emx1^ mice, impaired SC circuit responses could be attributed to abnormal dendritic development and decreased VGLUT1 expression [70], which are not affected in CPEB2-deficient neurons [35] (Fig. 2E). Because SC afferents synapsing onto the stratum radiatum of CA1 neurons comprise mostly a population of excitatory terminals that express VGLUT1 (Fig. 3A), increased PPF and decreased LTP in VGLUT2-cKO^Emx1^ mice resulted from decreased VGLUT1 level; whereas in CPEB2-cKO^Nes^ mice, they are likely caused by impaired translation of CPEB2-binding transcripts other than *Slc17a6* in SC afferents as well as reduced postsynaptic AMPAR level [35]. Although CPEB2 is expressed more predominantly in neurons than glia [79], astroglial transcripts, *Glul, Slc1a2* and *Slc1a3*, encoding glutamine synthetase and glutamate transporters, were also identified in CPEB2-immunoprecipitated transcriptome. Since astrocytes play a key role in glutamatergic transmission by controlling the glutamate-glutamine cycle and glutamate uptake in the synaptic cleft, further studies are needed to understand whether the loss of CPEB2 in astrocytes contributes to aberrant SC-CA1 transmission in CPEB2-cKO^Nes^ hippocampus [80]. To our knowledge, the TA-CA1 circuit has not been examined under the VGLUT2-deficient condition. VGLUT1 and VGLUT2 have the same function to control vesicular transport and synaptic release of glutamate [44, 64], so depletion of VGLUT2 in the TA-CA1 circuit is expected to cause similar electrophysiological defects. However, in this study, we identified that the synthesis of VGLUT2 but not VGLUT1 depends on CPEB2 (Fig. 2E). Moreover, VGLUT2 has a shorter half-life than VGLUT1 in cultured cortical neurons (Additional File 1: Fig. S8), implying that local translation may be more important for VGLUT2 than VGLUT1 to control glutamatergic plasticity.

CPEB1 can facilitate dendritic mRNA transport and activate NMDAR-dependent postsynaptic mRNA translation [28, 81], but the role of CPEBs in axonal mRNA transport and translation remains largely unexplored. Distinct from dendritic RNA granules, axonal mRNA is often co-transported with membrane-bound organelles, such as late endosomes and mitochondria, for the long-range delivery of mRNA molecules to distal axons [4]. Moreover, such a transport complex is an important regulon to link local translation and axonal function. For example, recent studies showed that *Pink1* mRNA, which encodes PTEN-induced putative kinase 1 (PINK1) for mitophagy of damaged mitochondria, is co-transported on mitochondria to axons, and its local translation is important to supply PINK1 for axonal mitophagy [82, 83]. Although axonal distribution of *Slc17a6* transcript was not affected in CPEB2-deficient TA afferents (Fig. 6C), further investigation is needed to determine whether CPEB2 may dispatch other identified mRNA candidates (Fig. 2C and Additional File 4) or co-transport with other membrane-bound organelles to axons. Interestingly, several mRNAs encoding transcriptional regulators, such as NF-*k*B and CREB-regulated transcription coactivator 1 (CRTC1), were identified in the learning-induced axonal translatome (Additional File 3). Since de novo transcription is also required to support long-lasting LTP and memory, activity-dependent nuclear transport of postsynaptically localized transcriptional regulators is a means of linking synaptic stimulation to transcriptional changes [84–87]. Nevertheless, whether such an activity-coupled nuclear transport mechanism exists in the presynaptic compartment of mature mammalian neurons and whether activity-regulated local translation contributes to the level of presynaptically resident transcriptional regulators to affect learning and memory remain to be explored.

Our reanalysis of learning-associated translatome (GSE124592) revealed 2,438 axon-specifically translated mRNAs, whereas Ostroff et al. found 454 transcripts. The duplicate TRAP experiments were conducted by using 2 different GFP antibodies to immunoprecipitate transcripts from eYFP-L10a (TRAP)- or YFP (control)-expressing cortical and amygdala tissues [20]. Because different antibodies can lead to different immunoprecipitation backgrounds, we chose to analyze the data separately rather than together as in the previous study [20]. *Slc17a6* was not identified previously because *Slc17a6* signals in the two TRAP experiments varied widely to cause a non-significant *P* value [20]. Nevertheless, activity-induced axonal translation of *Slc17a6* was experimentally demonstrated in this study, so the learning-associated axonal translatome appears to be more expanded and covers genes directly involved in presynaptic transmission. Besides CPEB2, FMR1 was recently shown to affect protein synthesis in hippocampal mossy fibers (MFs) and FMR1 deficiency impaired protein synthesis-dependent LTP in the MF-CA3 pathway [88], but which mRNAs are translationally regulated by FMR1 in MF afferents has not been identified. We therefore speculate that many other RBPs known to play a role in synaptic plasticity and memory may also be involved in presynaptic translation to build up the synaptic proteome for memory consolidation.

## Conclusions

This study identified a local translation mechanism contributing to the regulation of presynaptic plasticity. CPEB2-controlled translation governs presynaptic glutamatergic transmission and LTP in transected SC and TA afferents. CPEB2 binds to 272 mRNAs whose axonal translation changes after learning. Moreover, activity- and CPEB2-dependent axonal translation of VGLUT2 mRNA replenishes synaptic vesicles and sustains presynaptic translation-dependent LTP. These findings provide evidence linking local translational control in the regulation of glutamatergic transmission and presynaptic plasticity.

### Supplementary Information

Supplementary Material 1.

Supplementary Material 2.

Supplementary Material 3.

Supplementary Material 4.

Supplementary Material 5.

## Data Availability

Microarray data can be viewed by the GEO accession GSE208738: please access https://www.ncbi.nlm.nih.gov/geo/query/acc.cgi?acc=GSE208738 . Analyzed gene expression profiles from GSE208738, learning-associated translatome (GSE124592) and in combination are listed in Additional files 2, 3 and 4. All materials are available from the corresponding author upon reasonable request.

## Abbreviations

3′-UTR: 3′-Untreanslated region
4X HFS: 4 Trains of high-frequency stimulation
AAV: Adeno-associated virus
AMPAR: Alpha-amino-3-hydroxy-5-methyl-4-isoxazolepropionic acid-type glutamate receptor
APV: (2R)-amino-5-phosphonovaleric acid
BCA: Bicinchoninic acid
BSA: Bovine serum albumin
CA1: Cornu ammonis 1
CA3: Cornu ammonis 3
CaMK2α: Calcium/calmodulin-dependent protein kinase 2 subunit alpha
CHX: Cycloheximide
cKO: Conditional knockout
CNQX: Cyanquixaline
CPEB2: Cytoplasmic polyadenylation element binding protein 2
CRTC1: CREB regulated transcription coactivator 1
cWT: Conditional wild type
DEPC: Diethyl pyrocarbonate
DIV: Days in vitro
DTT: Dithiothreitol
EDTA: Ethylenediamine tetraacetic acid
EGTA: Ethylene glycol-bis(β-aminoethyl ether)-N,N,N′,N′-tetraacetic acid
EPM: Elevated plus maze
FISH: Fluorescence in situ hybridization
FMR1: Fragile X messenger ribonucleoprotein 1
fEPSP: Field excitatory postsynaptic potential
FV: Fiber volley
GFP: Green fluorescent protein
GO: Gene ontology
GRASP1: GRIP-associated protein 1
HEK293T: Human embryonic kidney 293 T
IRES: Internal ribosome entry site
KO: Knockout
LTP: Long-term potentiation
MF: Mossy fiber
MWM: Morris water maze
N2a: Neuro-2a
NMDA: N-methyl-D-aspartate
PBS: Phosphate buffered saline
PINK1: PTEN-induced putative kinase 1
PPF: Paired-pulse facilitation
RBP: RNA-binding protein
RIP: RNA immunoprecipitation
SC: Schaffer collateral
SLM: Stratum lacunosum moleculare
SM: Stratum moleculare
SO: Stratum oriens
SR: Stratum radiatum
SYT1: Synaptotagmin 1
TA: Temporoammonic
TRAP: Translating ribosome affinity purification
vg: Viral genome
VGLUT: Vesicular glutamate transporter
WT: Wild type
