# Supplementary material for: CPEB2-activated axonal translation of VGLUT2 mRNA promotes glutamatergic transmission and presynaptic plasticity
Source: J Biomed Sci. 2024 Jul 11;31:69. doi: 10.1186/s12929-024-01061-2 (PMC11241979; doi:10.1186/s12929-024-01061-2)
Supplement: Supplementary file 1 — Supplementary Material 1. [file 12929_2024_1061_MOESM1_ESM.pdf]

CPEB2-activated axonal translation of VGLUT2 mRNA promotes glutamatergic transmission and presynaptic plasticity

Wen-Hsin Lu, Tzu-Tung Chang, Yao-Ming Chang, Yi-Hsiang Liu, Chia-Hsuan Lin, Ching-Shu Suen, Ming-Jing Hwang and Yi-Shuian Huang*

Additional File 1

Supplemental Method

Table S1

Figures S1-S8

Supplemental Method

Behavioral Assays

All assays were conducted during 13:30-17:30 light phase by observers who did not know the genotype of the mice until the tests had been completed. Behavioral tasks were performed with 2- to 4-month-old male littermates following the previous protocols with modifications (1-3).

Open field: Each mouse was released into a corner of the arena and allowed to explore for 10 min. The recorded moving trace of each mouse was analyzed by using the TopScan system (CleverSys).

Elevated plus maze: The elevated plus maze (EPM) consisted of two open arms with 1 cm ledges and two enclosed arms with 15 cm walls. The maze was elevated to a height of 50 cm above the floor during the task. The mouse behaviors were recorded in a 5 min testing period and analyzed by using the TopScan system (CleverSys).

Morris water maze: mice were trained for the hidden platform version during acquisition, which consisted of 4 trials daily for 4 consecutive days. Mice were released to the MWM from all other 3 quadrants except the target quadrant and were allowed to stand on the platform for 15 sec before being transferred back to cages. The probe trial was performed on the 5th day in the water maze without a platform. For the visible MWM task, the escape platform marked by a flag was placed to measure the swimming ability and visual acuity of the mice. The maximal swimming duration for all trials was 1 min with a 15-min inter-trial interval. The trajectories of the mice were recorded and analyzed by using TrackMot (Singa Technology Corp.Corp., Taiwan).

1. Chao HW, Tsai LY, Lu YL, Lin PY, Huang WH, Chou HJ, et al. Deletion of CPEB3 enhances hippocampus-dependent memory via increasing expressions of PSD95 and NMDA receptors. J Neurosci. 2013;33(43):17008-22.
2. Lu WH, Yeh NH, Huang YS. CPEB2 Activates GRASP1 mRNA Translation and Promotes AMPA Receptor Surface Expression, Long-Term Potentiation, and Memory. Cell Rep. 2017;21(7):1783-94.
3. Tsai LY, Chang YW, Lin PY, Chou HJ, Liu TJ, Lee PT, et al. CPEB4 knockout mice exhibit normal hippocampus-related synaptic plasticity and memory. PLoS One. 2013;8(12):e84978.

Table S1 Antibodies and dyes used in the study.
The catalogue number and dilution information of antibodies and dyes.

Antigen	Host species	Company	Cat. No.	Clone	Dilution
CaMK2α	mouse	Merck	05-532	6G9	WB 1:500
CaMK2α-pThr286	mouse	Merck	05-533	22B1	WB 1:500
CPEB2	mouse	homemade	2aB4	monoclonal	WB/ICC 1:500<br>IHC 1:100<br>DAB 1:50
CPEB2	rabbit	homemade	7182W	polyclonal	ICC 1:20
GAPDH	mouse	Merck	MAB374	6C5	WB 1:1000
CaMK2α	rabbit	Cell Signaling	4436	polyclonal	IHC 1:200
LRP130	rabbit	Santa Cruz Biotechnology	sc-66845	polyclonal	WB 1:1000
MAP2	chicken	Novus Biologicals	NB300-213	polyclonal	IHC 1:500
mouse IgG	—	Merck	I5381	—	RIP
PSD95	mouse	Merck	MABN68	K28/43	WB 1:1000
synaptotagmin 1	rabbit	Synaptic Systems	105 008	Rb41.1	WB 1:500
Tau-1	mouse	Merck	MAB3420	PC1C6	ICC 1:200
βIII-tubulin	rabbit	ABclonal	A17074	polyclonal	ICC 1:500
VGLUT1	guinea pig	Merck	AB5905	polyclonal	WB 1:1000<br>IHC 1:500
VGLUT2	guinea pig	Merck	AB2251-I	polyclonal	WB/ICC 1:1000<br>IHC 1:500
guinea pig IgG, HRP	goat	Merck	AP108P	polyclonal	WB 1:10000
mouse IgG, HRP	goat	PerkinElmer	NEF822001EA	—	WB 1:10000
Rabbit IgG, HRP	goat	Jackson ImmunoResearch	111-035-003	polyclonal	WB 1:10000
Chicken IgY, Alexa Fluor™ 647	goat	Thermo Fisher Scientific	A-21449	polyclonal	IHC 1:1000
guinea pig IgG, Alexa Fluor™ 488	goat	Thermo Fisher Scientific	A-11073	polyclonal	ICC 1:2000<br>IHC 1:1000
mouse IgG, Alexa Fluor™ 594	donkey	Thermo Fisher Scientific	A-21203	polyclonal	IHC 1:200
mouse IgG, Alexa Fluor™ 647	donkey	Thermo Fisher Scientific	A-31571	polyclonal	ICC 1:1000
rabbit IgG, Alexa Fluor™ 405	goat	Thermo Fisher Scientific	A-31556	polyclonal	ICC 1:1000
rabbit IgG, Alexa Fluor™ 488	donkey	Thermo Fisher Scientific	A-21206	polyclonal	ICC 1:50
Hoechst 33342	—	Thermo Fisher Scientific	H3570	—	ICC/IHC 1:10000
FM™ 4-64FX	—	Thermo Fisher Scientific	F34653	—	10 μM

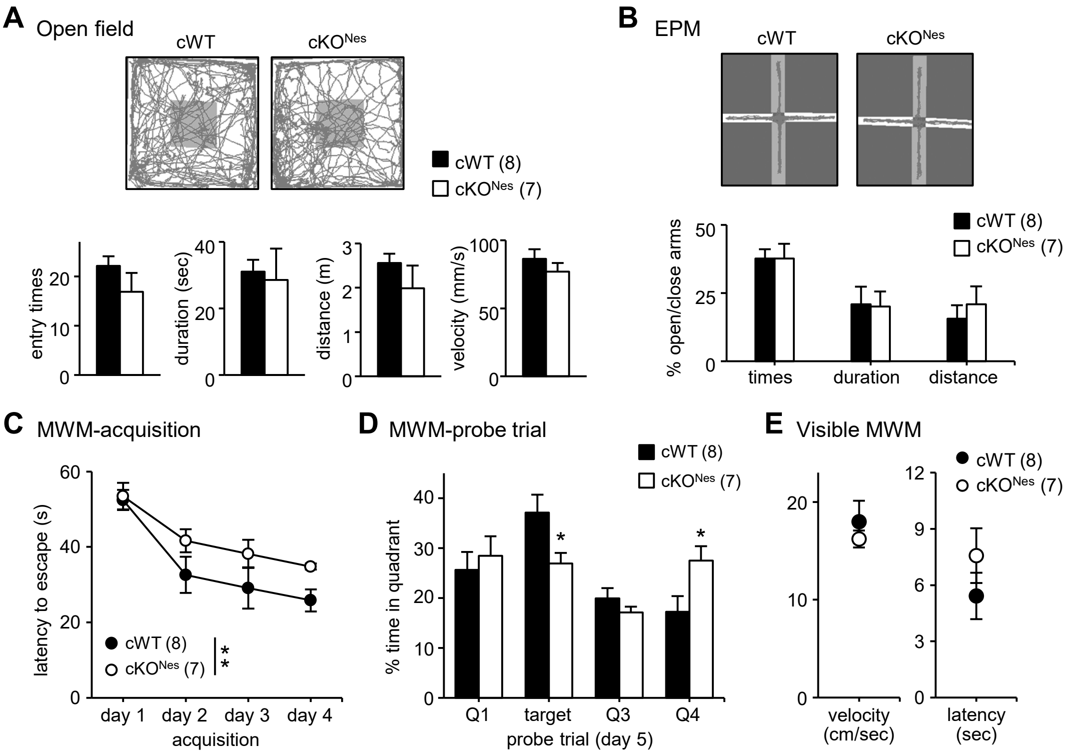

Fig. S1 Impaired spatial learning and memory in CPEB2-cKO^{Nes} mice.

Adult male mice at 2 to 4 months old were used for behavior assays. **A** Open-field. Representative moving traces in the open arena during the first 10 min and the quantified entry times, duration, moving distance and velocity. **B** Elevated plus maze (EPM). Representative 5-min moving traces in the EPM and the quantified entry times, duration and moving distance in the open versus closed arms. **C** Morris water maze (MWM). Mice were trained with 4 trials per day for 4 consecutive days, and the escape latency to the platform was averaged from 4 trials. **D** The MWM probe test on day 5 recorded the percentage of time (total 60 sec) spent in each quadrant. **E** In the visual MWM task, the velocity and latency reflected the swimming ability and visual acuity, respectively. Data are mean ± SEM. **P* < 0.05 and ***P* < 0.01, Student's *t* test and two-way ANOVA with Fisher's LSD *post-hoc* test. The number of mice for behavioral assays is in parentheses.

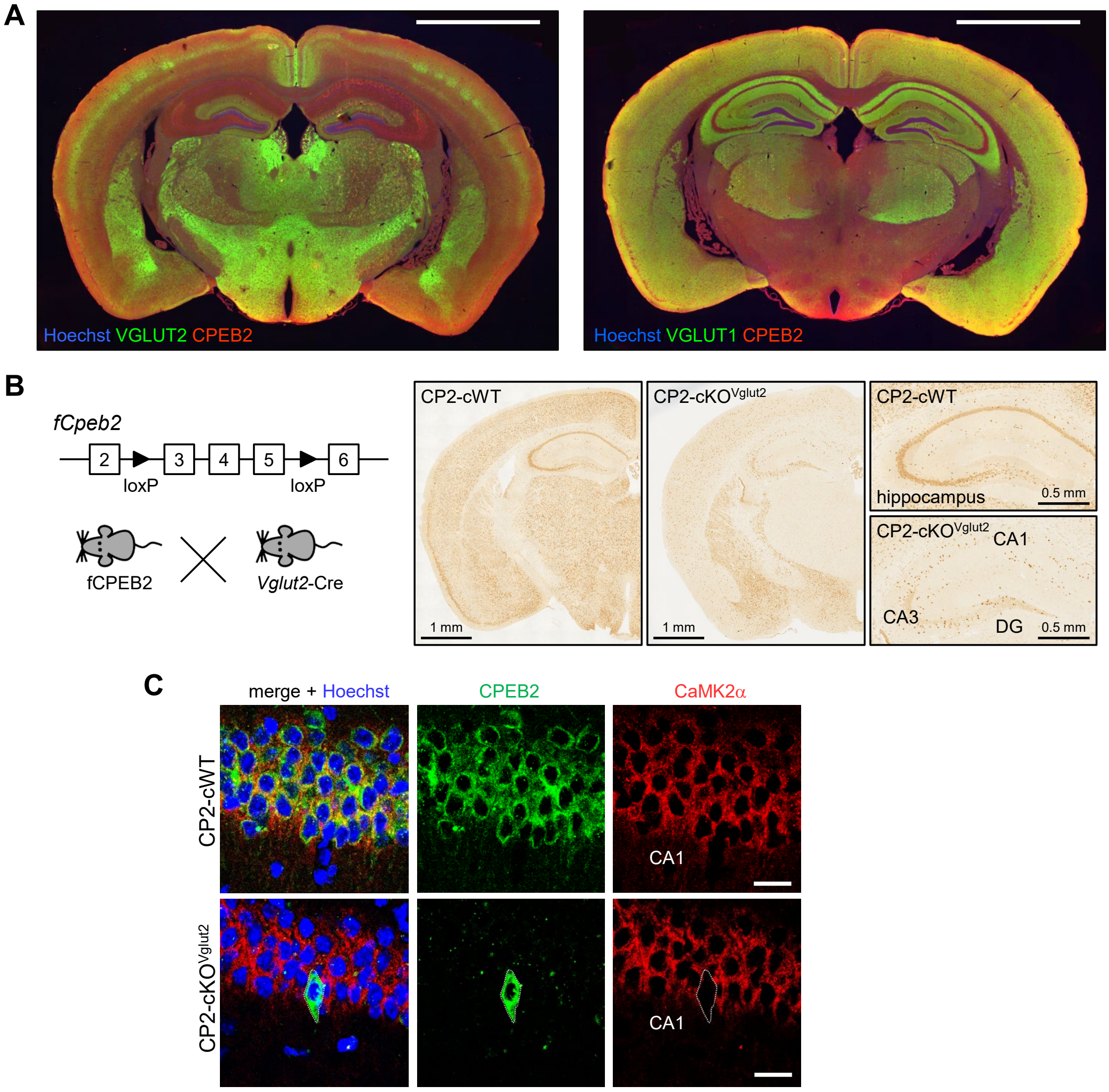

Fig. S2 The expression pattern of VGLUT2, VGLUT1 and CPEB2 in the adult mouse Brain.

A Coronal brain slices from adult mice were immunostained by using CPEB2 antibody and VGLUT2 or VGLUT1 antibody. Scales, 2 mm. **B** Mice carrying the *Cpeb2* allele with two loxP sites flanking exons 3-5 (fCPEB2) were crossed with *Vglut2*-Cre mice to generate CPEB2-cWT and -cKO^{Vglut2} mice. Coronal brain slices prepared from adult CPEB2-cWT and -cKO^{Vglut2} mice were used for immunohistochemistry of CPEB2 or **C** immunofluorescence staining of CPEB2 and CaMK2α. Scales, 20 μm

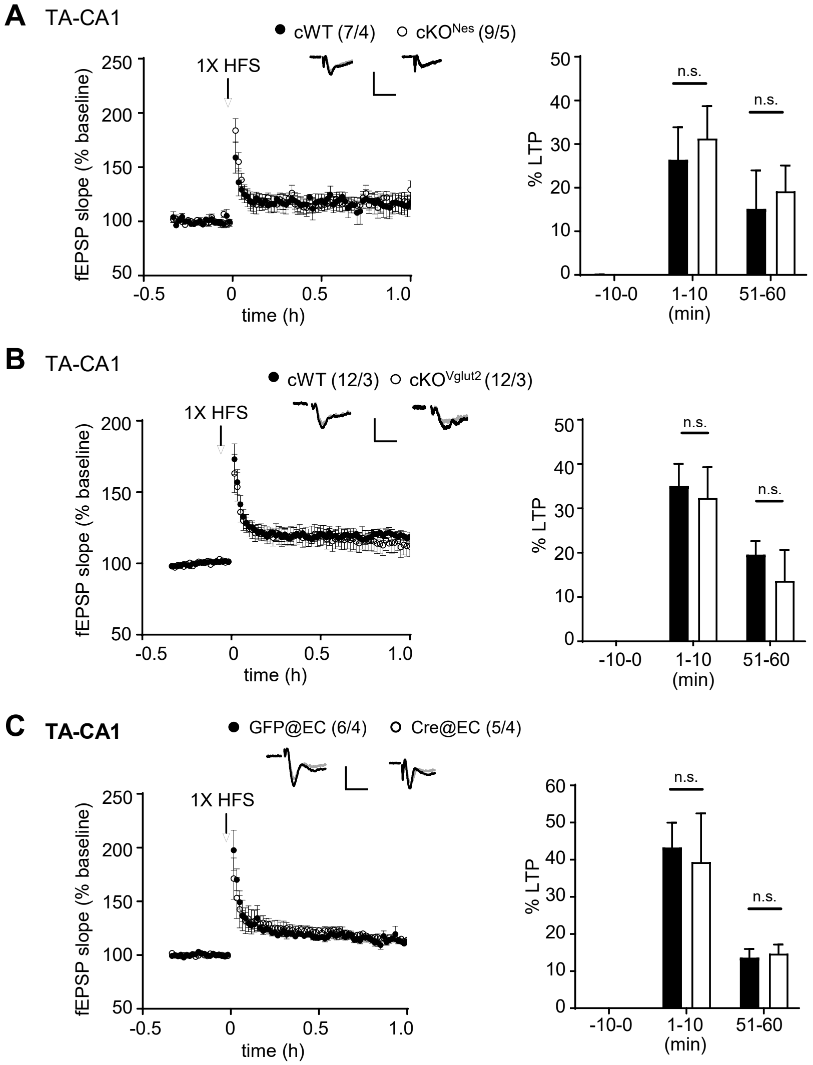

Fig. S3 Normal 1X HFS-stimulated LTP in CPEB2-deficient TA-CA1 pathway.
A CPEB2-cKO^{Nes} and **B** CPEB2-cKO^{Vglut2} male mice (3 to 5 months old) were used for LTP recording induced by 1X HFS in the TA-CA1 circuit. **C** The same recording was performed in CPEB2-cWT male mice (4 to 5 months old) injected with AAV8-GFP (4.2 x 10^9 vg) or AAV9-Cre (1.62 x 10^{10} vg) at the lateral entorhinal cortex (@EC). Pre-synaptic deletion of CPEB2 had no effect on 1X HFS-evoked LTP in the TA-CA1 circuit. Numbers in parentheses (n/N) represent the number of recorded slices (n) and mice (N). Sample traces with vertical scales of 0.5 mV and horizontal scales of 10 ms presented in the same manner as described in Fig. 1. Data are mean ± SEM.

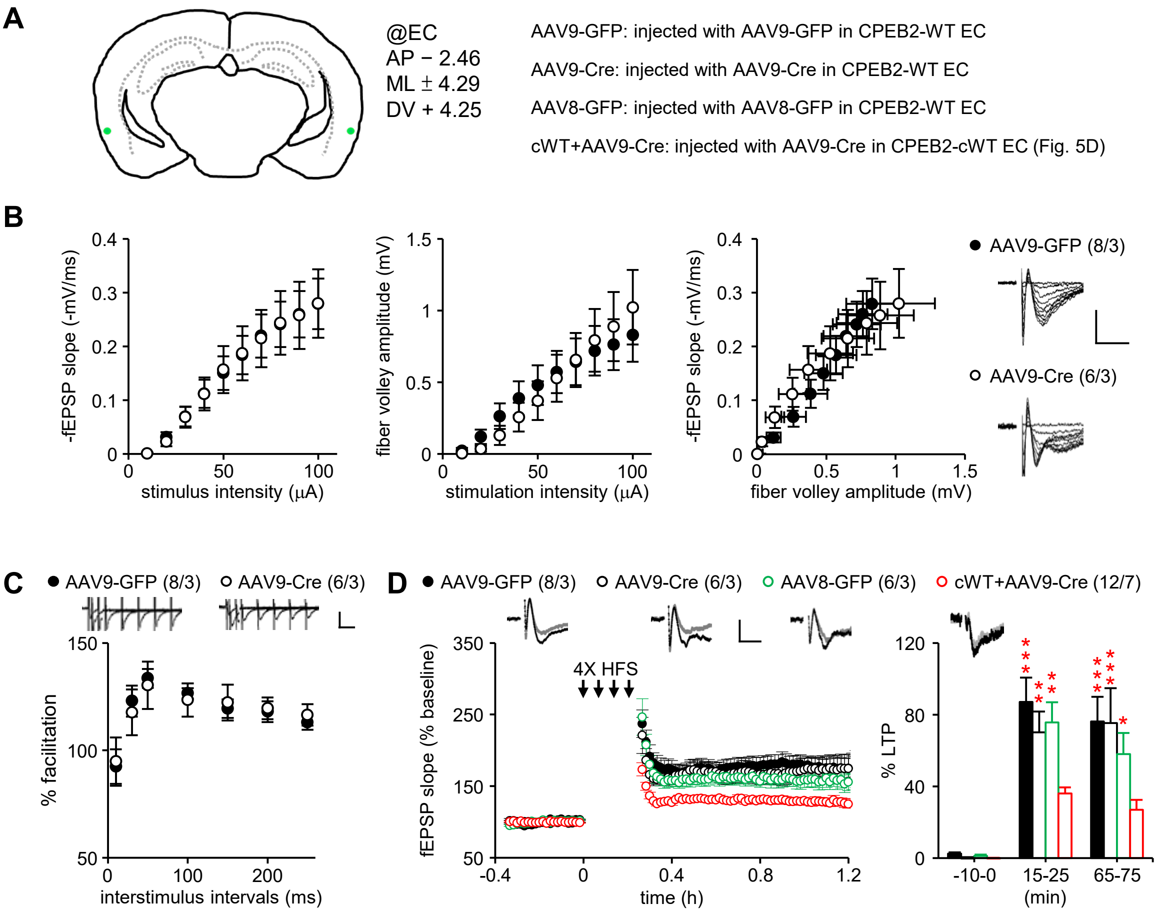

Fig. S4 No evident Cre-mediated effect on electrophysiological properties in the TA-CA1 pathway.

A AAV9-GFP (1.62 x 10^{10} vg), AAV9-Cre (1.62 x 10^{10} vg) or AAV8-GFP (4.2 x 10^{9} vg) were intracranially injected to the lateral entorhinal cortex (@EC, marked in green dots) of 5-month-old wild-type (WT) male mice. Approximately 3 weeks after intracranial delivery of AAV, hippocampal slices were used for field recording. **B** The input-output responses and **C** paired-pulse facilitation in the TA-CA1 pathway. **D** The 4X HFS-evoked LTP in WT mice injected with denoted viruses was compared to that in CPEB2-cWT mice injected with AAV9-Cre (data from Fig. 5D). Numbers in parentheses (n/N) represent the number of recorded slices (n) and mice (N). Sample traces were presented in the same manner as described in Fig 1. Data are mean ± SEM. $*P < 0.05$, $**P < 0.01$ and $***P < 0.001$, compared to the cWT+AAV9-Cre group, two-way ANOVA with Fisher's LSD *post hoc* test.

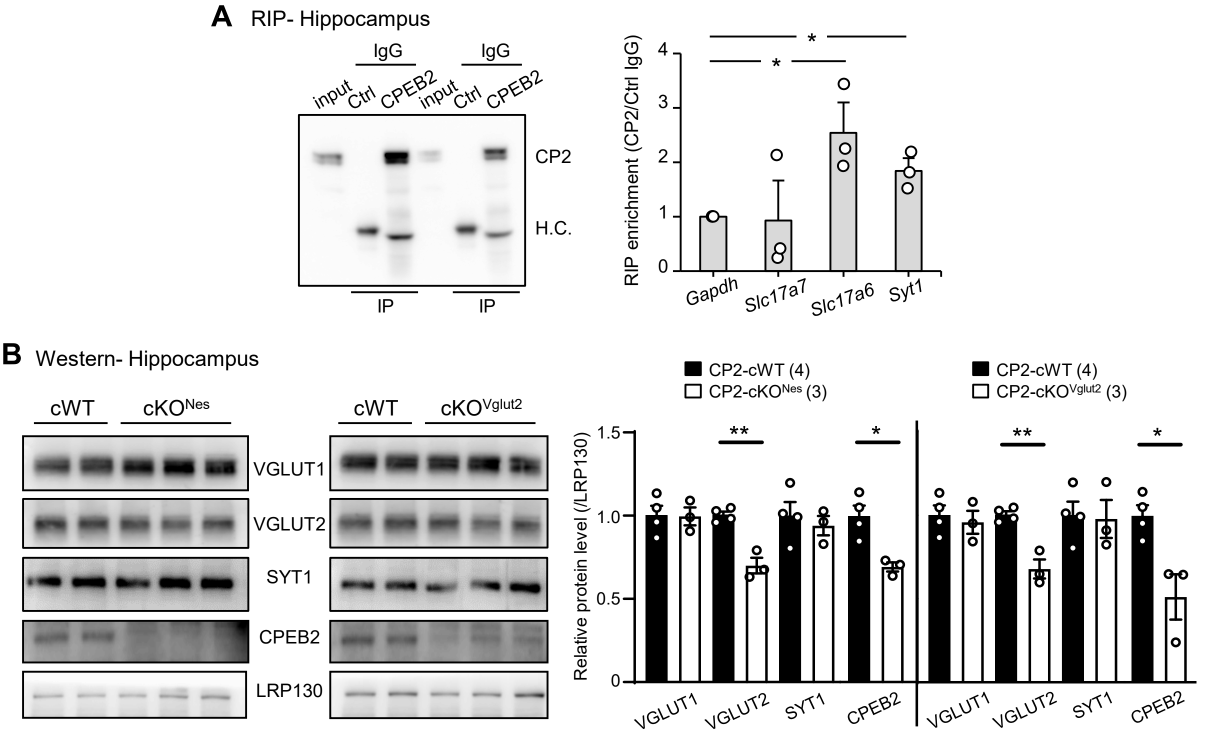

Fig. S5 CPEB2 bound to *Slc17a6* transcript and promoted VGLUT2 expression in the hippocampus.

A Adult mouse hippocampi were used for RNA immunoprecipitation (RIP) with CPEB2 or control (Ctrl) IgG. The precipitated substances were processed for western blot analysis or RNA isolation for RT-qPCR for level of *Slc17a7*, *Slc17a6* and *Syt1* mRNA with the non-target control, *Gapdh* mRNA, as the reference. Data are mean ± SEM from 3 independent experiments. **B** Adult hippocampi from CPEB2-cKO^{Nes} and CPEB2-cKO^{Vglut2} mice and their cWT littermates were used for immunoblotting. The protein levels were normalized to that of LRP130. The number of mice is in parentheses. Data are mean ± SEM. *$P < 0.05$ and **$P < 0.01$, Student's *t* test.

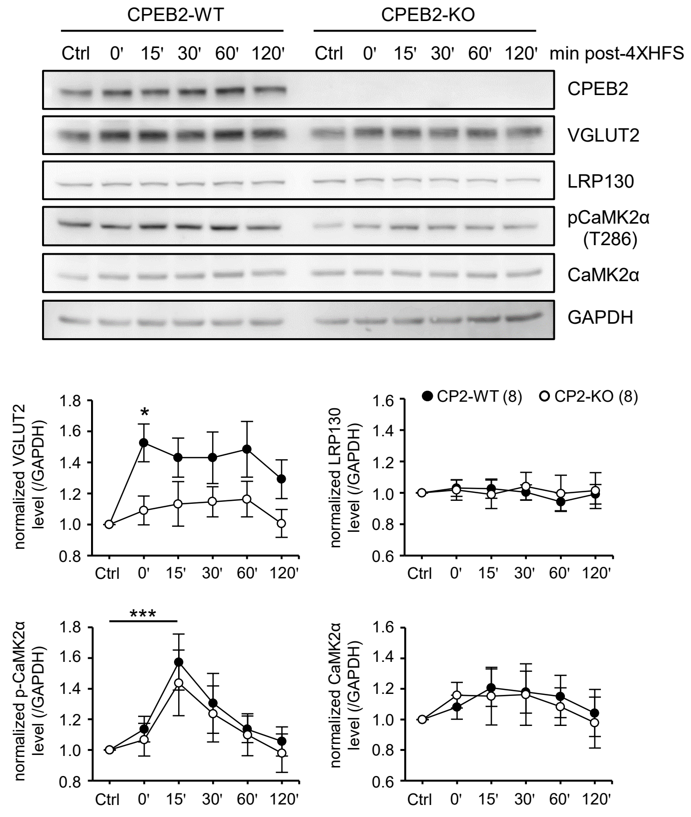

Fig. S6 4X HFS increased VGLUT2 expression in CPEB2-WT but not CPEB2-KO neurons.

DIV20-25 neurons were challenged without (Ctrl) or with 4X HFS, then harvested at the indicated times for immunoblotting. The levels of VGLUT2, LRP130, p-CaMK2α and CaMK2α were normalized to that of GAPDH. Data are mean ± SEM from 8 experiments from 6 independent cultures. *$P < 0.05$ and ***$P < 0.001$, two-way ANOVA with Fisher's LSD *post hoc* test.

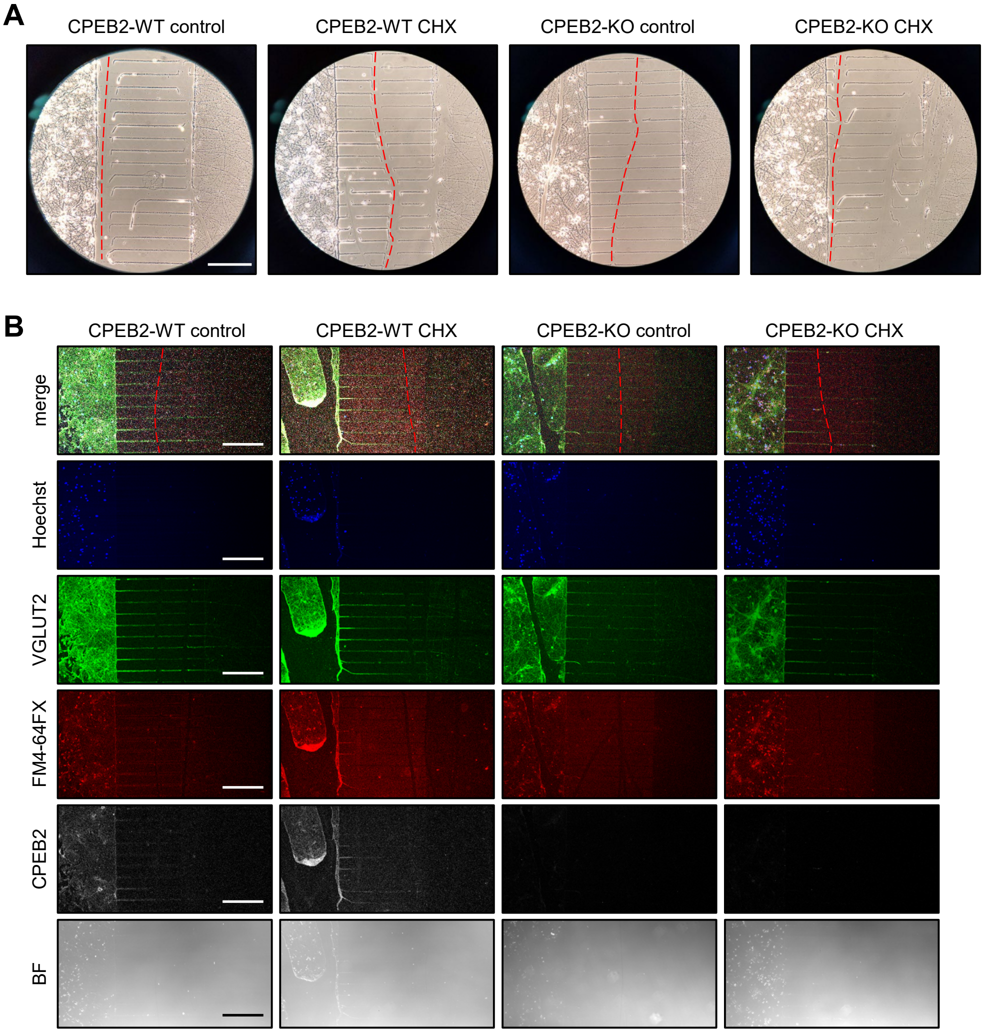

Fig. S7 Axotomy applied to neurons grown in microfluidic chambers.
A DIV18-19 CPEB2-WT and -KO neurons cultured on microfluidic chambers after axotomy (marked by red dashed lines) were stimulated with 4X HFS and incubated for 2 h ± CHX. **B** After 2 h incubation, axotomized neurons in Tyrode's buffer containing 10 µM FM4-64FX, 50 µM APV and 10 µM CNQX were stimulated with 10 Hz for 90 sec, then incubated for 20 min followed by 10 min in 1 mM ADVASEP-7, 50 µM APV and 10 µM CNQX before fixation and immunostaining of CPEB2 and VGLUT2. Representative images showed somas on the left side and axonal projections on the right side. Scale, 0.2 mm.

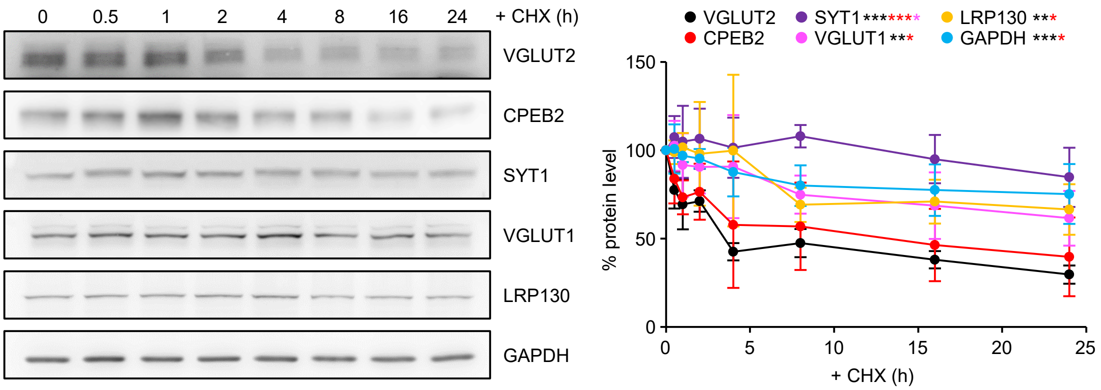

Fig. S8 VGLUT2 was less stable than VGLUT1 and SYT1 in cultured neurons.
DIV20 rat cortical neurons were treated with 20 µg/ml cycloheximide (CHX) to block new protein synthesis for the indicated times and then harvested for immunoblotting of denoted proteins. The amount of individual protein was expressed as a relative percentage to the time zero which was arbitrarily set to 100. Data are mean ± SEM from 3 independent cultures. *$P < 0.05$, **$P < 0.01$ and ***$P < 0.001$, two-way ANOVA.
