# Supplementary material for: CPEB2-activated axonal translation of VGLUT2 mRNA promotes glutamatergic transmission and presynaptic plasticity
Source: J Biomed Sci. 2024 Jul 11;31:69. doi: 10.1186/s12929-024-01061-2 (PMC11241979; doi:10.1186/s12929-024-01061-2)
Supplement: Supplementary file 5 — Supplementary Material 5. [file 12929_2024_1061_MOESM5_ESM.docx]

Statistical Table

| **Figure** | **Sample** | **Type of test** | **p-values** |
| --- | --- | --- | --- |
| Fig 1B  fEPSP slope | stimulus intensity  cWT v.s. cKO^Nes^ | Two-way ANOVA  F_(9,160)_ = 5.652  F_(1,160)_ = 32.264 | < 0.001 ***  < 0.001 *** |
| Fig 1B  fEPSP slope  cWT v.s. cKO^Nes^ | 10 μA  20 μA  30 μA  40 μA  50 μA  60 μA  70 μA  80 μA  90 μA  100 μA | Fisher's LSD *post hoc* test | 0.992  0.613  0.184  0.072  0.030 *  0.014 *  0.012 *  0.014 *  0.032 *  0.016 * |
| Fig 1B  fiber volley amplitude | stimulus intensity  cWT v.s. cKO^Nes^ | Two-way ANOVA  F_(9,160)_ = 10.397  F_(1,160)_ = 9.928 | < 0.001 ***  0.002 ** |
| Fig 1B  fiber volley amplitude  cWT v.s. cKO^Nes^ | 10 μA  20 μA  30 μA  40 μA  50 μA  60 μA  70 μA  80 μA  90 μA  100 μA | Fisher's LSD *post hoc* test | 0.343  0.952  0.865  0.953  0.852  0.429  0.263  0.043 *  0.014 *  0.004 ** |
| Fig 1C  paired-pulse facilitation | interstimulus intervals  cWT v.s. cKO^Nes^ | Two-way ANOVA  F_(6,154)_ = 5.720  F_(1,154)_ = 27.033 | < 0.001 ***  < 0.001 *** |
| Fig 1C  paired-pulse facilitation  cWT v.s. cKO^Nes^ | 10 ms  30 ms  50 ms  100 ms  150 ms  200 ms  250 ms | Fisher's LSD *post hoc* test | 0.009 **  0.059  0.018 *  0.041 *  0.042 *  0.138  0.227 |
| Fig 1D  4X HFS | min  cWT v.s. cKO^Nes^ | Two-way ANOVA  F_(3,40)_ = 0.922  F_(1,40)_ = 15.149 | 0.439  < 0.001 *** |
| Fig 1D  4X HFS  cWT v.s. cKO^Nes^ | 15-25 min  50-60 min  110-120 min  170-180min | Fisher's LSD *post hoc* test | 0.095  0.159  0.020 *  0.032 * |
| Fig 2E  adult cortex | VGLUT2  SYT1  VGLUT1 | Mann-Whitney rank sum test  T = 57.000  T = 39.000  T = 33.000 | 0.002 **  1.000  0.394 |
| Fig 2E  DIV21 neuron | VGLUT2  SYT1  VGLUT1 | Mann-Whitney rank sum test  T = 57.000  T = 39.000  T = 45.000 | 0.002 **  1.000  0.394 |
| Fig 2F | adult cortex  DIV21 neuron | Mann-Whitney rank sum test  T = 51.000  T = 45.000 | 0.065  0.394 |
| Fig 2G | HEK293T  N2a | One-way ANOVA / Kruskal-Wallis one-way ANOVA  F_(2,15)_ = 6.150  H = 22.338 | 0.011 *  < 0.001 *** |
| Fig 2G  HEK293T | EGFP v.s. myc-CP2  EGFP v.s. myc-CP2C  myc-CP2 v.s. myc-CP2C | Fisher's LSD *post hoc* test | 0.033 *  0.299  0.004 ** |
| Fig 2G  N2a | EGFP v.s. myc-CP2  EGFP v.s. myc-CP2C  myc-CP2 v.s. myc-CP2C | Tukey test / t-test | <0.001 ***  0.003 **  < 0.001 *** |
| Fig 3C  fEPSP slope | stimulus intensity  cWT v.s. cKO^Nes^ | Two-way ANOVA  F_(9,260)_ = 11.915  F_(1,260)_ = 58.060 | < 0.001 ***  < 0.001 *** |
| Fig 3C  fEPSP slope  cWT v.s. cKO^Nes^ | 10 μA  20 μA  30 μA  40 μA  50 μA  60 μA  70 μA  80 μA  90 μA  100 μA | Fisher's LSD *post hoc* test | 0.806  0.505  0.213  0.106  0.023 *  0.003 **  0.001 **  < 0.001 ***  < 0.001 ***  < 0.001 *** |
| Fig 3C  fiber volley amplitude | stimulus intensity  cWT v.s. cKO^Nes^ | Two-way ANOVA  F_(9,260)_ = 7.291  F_(1,260)_ = 17.149 | < 0.001 ***  < 0.001 *** |
| Fig 3C  fiber volley amplitude  cWT v.s. cKO^Nes^ | 10 μA  20 μA  30 μA  40 μA  50 μA  60 μA  70 μA  80 μA  90 μA  100 μA | Fisher's LSD *post hoc* test | 0.845  0.606  0.696  0.685  0.453  0.345  0.071  0.041 *  0.005 **  0.002 ** |
| Fig 3D  paired-pulse facilitation | interstimulus intervals  cWT v.s. cKO^Nes^ | Two-way ANOVA  F_(6,161)_ = 3.207  F_(1,161)_ = 19.319 | 0.005 **  < 0.001 *** |
| Fig 3D  paired-pulse facilitation  cWT v.s. cKO^Nes^ | 10 ms  30 ms  50 ms  100 ms  150 ms  200 ms  250 ms | Fisher's LSD *post hoc* test | 0.106  0.058  0.095  0.129  0.029 *  0.225  0.144 |
| Fig 3E  4X HFS | min  cWT v.s. cKO^Nes^ | Two-way ANOVA  F_(2,51)_ = 5.372  F_(1,51)_ = 8.771 | 0.008 **  0.005 ** |
| Fig 3E  4X HFS  cWT v.s. cKO^Nes^ | -10-0 min  15-25 min  65-75min | Fisher's LSD *post hoc* test | 0.782  0.047 *  0.007 ** |
| Fig 3F  fEPSP slope | stimulus intensity  cWT v.s. cKO^Vglut2^ | Two-way ANOVA  F_(9,200)_ = 15.043  F_(1,200)_ = 35.106 | < 0.001 ***  < 0.001 *** |
| Fig 3F  fEPSP slope  cWT v.s. cKO^Vglut2^ | 10 μA  20 μA  30 μA  40 μA  50 μA  60 μA  70 μA  80 μA  90 μA  100 μA | Fisher's LSD *post hoc* test | 0.769  0.453  0.278  0.097  0.034 *  0.029 *  0.017 *  0.015 *  0.008 **  0.003 ** |
| Fig 3F  fiber volley amplitude | stimulus intensity  cWT v.s. cKO^Vglut2^ | Two-way ANOVA  F_(9,200)_ = 7.39  F_(1,200)_ = 25.076 | < 0.001 ***  < 0.001 *** |
| Fig 3F  fiber volley amplitude  cWT v.s. cKO^Vglut2^ | 10 μA  20 μA  30 μA  40 μA  50 μA  60 μA  70 μA  80 μA  90 μA  100 μA | Fisher's LSD *post hoc* test | 0.649  0.399  0.151  0.083  0.054  0.045 *  0.094  0.060  0.069  0.047 * |
| Fig 3G  paired-pulse facilitation | interstimulus intervals  cWT v.s. cKO^Vglut2^ | Two-way ANOVA  F_(6,133)_ = 4.823  F_(1,133)_ = 12.826 | < 0.001 ***  < 0.001 *** |
| Fig 3G  paired-pulse facilitation  cWT v.s. cKO^Vglut2^ | 10 ms  30 ms  50 ms  100 ms  150 ms  200 ms  250 ms | Fisher's LSD *post hoc* test | 0.515  0.009 **  0.037 *  0.144  0.647  0.764  0.067 |
| Fig 3H  4X HFS | min  cWT v.s. cKO^Vglut2^ | Two-way ANOVA  F_(2,42)_ = 13.041  F_(1,42)_ = 11.022 | < 0.001 ***  0.002 ** |
| Fig 3H 4X HFS  cWT v.s. cKO^Vglut2^ | -10-0 min  15-25 min  65-75min | Fisher's LSD *post hoc* test | 0.963  < 0.001 ***  0.043 * |
| Fig 4A | entry times  duration  distance  velocity | Mann-Whitney rank sum test  T = 62.500  T = 57.000  T = 61.500  T = 68.000 | 0.463  0.955  0.536  0.189 |
| Fig 4B | times  duration  distance | t-test  t = 1.865  t = 0.140  t = 1.202 | 0.085  0.891  0.251 |
| Fig 4C  MWM-acquisition | genotype  days | Two-way ANOVA  F_(1,52)_ = 2.527  F_(3,52)_ = 17.818 | 0.118  < 0.001 *** |
| Fig 4C  MWM-acquisition  cWT v.s. cKO^Vglut2^ | day 1  day 2  day 3  day 4 | Fisher's LSD *post hoc* test | 0.715  0.217  0.274  0.651 |
| Fig 4D  MWM-probe trial | genotype * quadrant | Two-way ANOVA  F_(3,52)_ = 4.127 | 0.011 * |
| Fig 4D  MWM-probe trial  cWT v.s. cKO^Vglut2^ | Q1  target  Q3  Q4 | Fisher's LSD *post hoc* test | 0.351  0.006 **  0.970  0.069 |
| Fig 4E  Visible MWM | velocity  latency | t-test  t = 0.547  t = 0.157 | 0.594  0.877 |
| Fig 5B  fEPSP slope | stimulus intensity  GFP@EC v.s. Cre@EC | Two-way ANOVA  F_(9,180)_ = 38.721  F_(1,180)_ = 0.479 | < 0.001 ***  0.490 |
| Fig 5B  fiber volley amplitude | stimulus intensity  GFP@EC v.s. Cre@EC | Two-way ANOVA  F_(9,180)_ = 30.482  F_(1,180)_ = 6.095 | < 0.001 ***  0.014 * |
| Fig 5B  fiber volley amplitude  GFP@EC v.s. Cre@EC | 10 μA  20 μA  30 μA  40 μA  50 μA  60 μA  70 μA  80 μA  90 μA  100 μA | Fisher's LSD *post hoc* test | 0.956  0.846  0.916  0.676  0.578  0.329  0.184  0.200  0.134  0.039 * |
| Fig 5C  paired-pulse facilitation | interstimulus intervals  GFP@EC v.s. Cre@EC | Two-way ANOVA  F_(6,126)_ = 8.301  F_(1,126)_ = 12.933 | < 0.001 ***  < 0.001 *** |
| Fig 5C  paired-pulse facilitation  GFP@EC v.s. Cre@EC | 10 ms  30 ms  50 ms  100 ms  150 ms  200 ms  250 ms | Fisher's LSD *post hoc* test | 0.036 *  0.684  0.094  0.139  0.093  0.178  0.444 |
| Fig 5D  4X HFS | min  GFP@EC v.s. Cre@EC | Two-way ANOVA  F_(2,54)_ = 40.319  F_(1,54)_ = 12.656 | < 0.001 ***  < 0.001 *** |
| Fig 5D  4X HFS  GFP@EC v.s. Cre@EC | -10-0 min  15-25 min  65-75min | Fisher's LSD *post hoc* test | 0.845  < 0.001 ***  0.016 * |
| Fig 5E  fEPSP slope | stimulus intensity  GFP@CA1 v.s. Cre@CA1 | Two-way ANOVA  F_(9,140)_ = 24.044  F_(1,140)_ = 15.193 | < 0.001 ***  < 0.001 *** |
| Fig 5E  fEPSP slope  GFP@CA1 v.s. Cre@CA1 | 10 μA  20 μA  30 μA  40 μA  50 μA  60 μA  70 μA  80 μA  90 μA  100 μA | Fisher's LSD *post hoc* test | 0.883  0.998  0.837  0.484  0.158  0.072  0.062  0.073  0.024 *  0.020 * |
| Fig 5E  fiber volley amplitude | stimulus intensity  GFP@CA1 v.s. Cre@CA1 | Two-way ANOVA  F_(9,140)_ = 13.079  F_(1,140)_ = 0.938 | < 0.001 ***  0.334 |
| Fig 5F  paired-pulse facilitation | interstimulus intervals  GFP@CA1 v.s. Cre@CA1 | Two-way ANOVA  F_(6,98)_ = 9.339  F_(1,98)_ = 0.0823 | < 0.001 ***  0.775 |
| Fig 5G  4X HFS | min  GFP@CA1 v.s. Cre@CA1 | Two-way ANOVA  F_(2,42)_ = 25.193  F_(1,42)_ = 6.289 | < 0.001 ***  0.016 * |
| Fig 5D  4X HFS  GFP@CA1 v.s. Cre@CA1 | -10-0 min  15-25 min  65-75min | Fisher's LSD *post hoc* test | 0.883  0.155  0.009 ** |
| Fig 6B | axon v.s. soma | Mann-Whitney rank sum test  T = 122.000 | 0.112 |
| Fig 6C | cWT v.s. cKO^Nes^ | Normality test (Shapiro-Wilk)  t = 0.381 | 0.710 |
| Fig 6D  VGLUT2 | genotype  treatment | Two-way ANOVA  F_(1,70)_ = 6.562  F_(4,70)_ = 2.764 | 0.013 *  0.034 * |
| Fig 6D  VGLUT2  CP2-WT v.s. CP2-KO | Ctrl  15’  30’  60’  CHX | Fisher's LSD *post hoc* test | 1.000  0.040 *  0.120  0.101  0.694 |
| Fig 6D  VGLUT2  CP2-WT | Ctrl v.s. 15’  Ctrl v.s. 30’  Ctrl v.s. 60’  Ctrl v.s. CHX  15’ v.s. 30’  15’ v.s. 60’  15’ v.s. CHX  30’ v.s. 60’  30’ v.s. CHX  60’ v.s. CHX | Fisher's LSD *post hoc* test | 0.005 **  0.049 *  0.028 *  0.796  0.376  0.514  0.010 *  0.815  0.086  0.052 |
| Fig 6D  VGLUT2  CP2-KO | Ctrl v.s. 15’  Ctrl v.s. 30’  Ctrl v.s. 60’  Ctrl v.s. CHX  15’ v.s. 30’  15’ v.s. 60’  15’ v.s. CHX  30’ v.s. 60’  30’ v.s. CHX  60’ v.s. CHX | Fisher's LSD *post hoc* test | 0.427  0.670  0.567  0.892  0.712  0.824  0.353  0.883  0.575  0.479 |
| Fig 6D  PSD95 | genotype  treatment | Two-way ANOVA  F_(1,70)_ = 0.613  F_(4,70)_ = 0.0719 | 0.436  0.990 |
| Fig 6D  pCaMK2α | genotype  treatment | Two-way ANOVA  F_(1,70)_ = 0.849  F_(4,70)_ = 7.707 | 0.360  < 0.001 *** |
| Fig 6D  pCaMK2α  CP2-WT v.s. CP2-KO | Ctrl  15’  30’  60’  CHX | Fisher's LSD *post hoc* test | 1.000  0.721  0.581  0.397  0.770 |
| Fig 6D  pCaMK2α  CP2-WT | Ctrl v.s. 15’  Ctrl v.s. 30’  Ctrl v.s. 60’  Ctrl v.s. CHX  15’ v.s. 30’  15’ v.s. 60’  15’ v.s. CHX  30’ v.s. 60’  30’ v.s. CHX  60’ v.s. CHX | Fisher's LSD *post hoc* test | 0.002 **  0.015 *  0.260  0.869  0.435  0.036 *  0.003 **  0.179  0.023 *  0.336 |
| Fig 6D  pCaMK2α  CP2-KO | Ctrl v.s. 15’  Ctrl v.s. 30’  Ctrl v.s. 60’  Ctrl v.s. CHX  15’ v.s. 30’  15’ v.s. 60’  15’ v.s. CHX  30’ v.s. 60’  30’ v.s. CHX  60’ v.s. CHX | Fisher's LSD *post hoc* test | 0.005 **  0.057  0.778  0.899  0.330  0.010 *  0.003 **  0.102  0.043 *  0.683 |
| Fig 6D  CaMK2 | genotype  treatment | Two-way ANOVA  F_(1,70)_ = 0.0846  F_(4,70)_ = 0.767 | 0.772  0.550 |
| Fig 7A | genotype  treatment | Two-way ANOVA  F_(1,36)_ = 4.819  F_(1,36)_ = 2.585 | 0.035 *  0.117 |
| Fig 7A  CP2-WT v.s. CP2-KO | basal  4XHFS | Fisher's LSD *post hoc* test | 0.775  0.008 ** |
| Fig 7A  basal v.s. 4XHFS | CP2-WT  CP2-KO | Fisher's LSD *post hoc* test | 0.022 *  0.899 |
| Fig 7B  VGLUT2^+^ vesicles | genotype  treatment | Two-way ANOVA  F_(1,76)_ = 5.938  F_(1,76)_ = 11.546 | 0.017 *  0.001 ** |
| Fig 7B VGLUT2^+^ vesicles  CP2-WT v.s. CP2-KO | control  CHX | Fisher's LSD *post hoc* test | < 0.001 ***  0.889 |
| Fig 7B VGLUT2^+^ vesicles  control v.s. CHX | CP2-WT  CP2-KO | Fisher's LSD *post hoc* test | < 0.001 ***  0.591 |
| Fig 7B  VGLUT2^-^ vesicles | genotype  treatment | Two-way ANOVA  F_(1,76)_ = 0.00172  F_(1,76)_ = 0.886 | 0.967  0.350 |
| Fig 7C number of  VGLUT2^+^ vesicles | genotype  treatment | Two-way ANOVA  F_(1,76)_ = 14.306  F_(1,76)_ = 5.968 | < 0.001 ***  0.017 * |
| Fig 7C number of  VGLUT2^+^ vesicles  CP2-WT v.s. CP2-KO | control  CHX | Fisher's LSD *post hoc* test | 0.001 **  0.052 |
| Fig 7C number of  VGLUT2^+^ vesicles  control v.s. CHX | CP2-WT  CP2-KO | Fisher's LSD *post hoc* test | 0.018 *  0.307 |
| Fig 7C  VGLUT2^+^ intensity | genotype  treatment | Two-way ANOVA  F_(1,76)_ = 0.243  F_(1,76)_ = 0.779 | 0.624  0.380 |
| Fig 7C  VGLUT2^+^ area | genotype  treatment | Two-way ANOVA  F_(1,76)_ = 2.077  F_(1,76)_ = 3.323 | 0.154  0.072 |
| Fig S1A | entry times  duration  distance  velocity | t-test / Mann-Whitney rank sum test  t = 1.256  T = 45.000  t = 1.092  t = 0.993 | 0.231  0.232  0.295  0.339 |
| Fig S1B | times  duration  distance | t-test / Mann-Whitney rank sum test  t = 0.0024  T = 61.000  T = 60.000 | 0.998  0.613  0.694 |
| Fig S1C  MWM-acquisition | genotype  days | Two-way ANOVA  F_(1,52)_ = 7.173  F_(3,52)_ = 14.609 | 0.010 *  < 0.001 *** |
| Fig S1C  MWM-acquisition  cWT v.s. cKO^Nes^ | day 1  day 2  day 3  day 4 | Fisher's LSD *post hoc* test | 0.844  0.090  0.090  0.094 |
| Fig S1D  MWM-probe trial | genotype * quadrant | Two-way ANOVA  F_(3,52)_ = 4.182 | 0.010 * |
| Fig S1D  MWM-probe trial  cWT v.s. cKO^Nes^ | Q1  target  Q3  Q4 | Fisher's LSD *post hoc* test | 0.508  0.020 *  0.502  0.019 * |
| Fig S1E  Visible MWM | velocity  latency | t-test / Mann-Whitney rank sum test  t = 0.713  T = 68.000 | 0.489  0.189 |
| Fig S3A  TA pathway  1X HFS | min  cWT v.s. cKO^Nes^ | Two-way ANOVA  F_(2,42)_ = 11.342  F_(1,42)_ = 0.332 | < 0.001 ***  0.568 |
| Fig S3B  TA pathway  1X HFS | min  cWT v.s. cKO^Vglut2^ | Two-way ANOVA  F_(2,66)_ = 24.426  F_(1,66)_ = 0.567 | < 0.001 ***  0.454 |
| Fig S3C  TA pathway  1X HFS | min  cWT v.s. cKO^EC^ | Two-way ANOVA  F_(2,27)_ = 25.328  F_(1,27)_ = 0.0361 | < 0.001 ***  0.851 |
| Fig S4B  fEPSP slope | stimulus intensity  AAV9-GFP v.s. AAV9-Cre | Two-way ANOVA  F_(9,120)_ = 13.363  F_(1,120)_ = 0.000143 | < 0.001 ***  0.990 |
| Fig S4B  fEPSP slope  AAV9-GFP v.s. AAV9-Cre | 10 μA  20 μA  30 μA  40 μA  50 μA  60 μA  70 μA  80 μA  90 μA  100 μA | Fisher's LSD *post hoc* test | 0.979  0.881  0.987  0.990  0.906  0.951  0.931  0.961  0.968  0.989 |
| Fig S4B  fiber volley amplitude | stimulus intensity  AAV9-GFP v.s. AAV9-Cre | Two-way ANOVA  F_(9,120)_ = 8.96  F_(1,120)_ = 0.0285 | < 0.001 ***  0.866 |
| Fig S4B  fiber volley amplitude  AAV9-GFP v.s. AAV9-Cre | 10 μA  20 μA  30 μA  40 μA  50 μA  60 μA  70 μA  80 μA  90 μA  100 μA | Fisher's LSD *post hoc* test | 0.932  0.695  0.531  0.538  0.610  0.843  0.953  0.733  0.554  0.366 |
| Fig S4C  paired-pulse facilitation | interstimulus intervals  AAV9-GFP v.s. AAV9-Cre | Two-way ANOVA  F_(6,84)_ = 6.094  F_(1,84)_ = 0.00231 | < 0.001 ***  0.962 |
| Fig S4C  paired-pulse facilitation  AAV9-GFP v.s. AAV9-Cre | 10 ms  30 ms  50 ms  100 ms  150 ms  200 ms  250 ms | Fisher's LSD *post hoc* test | 0.809  0.591  0.741  0.757  0.773  0.871  0.719 |
| Fig S4D  4X HFS | min  between viruses | Two-way ANOVA  F_(2,84)_ = 64.827  F_(3,84)_ = 11.388 | < 0.001 ***  < 0.001 *** |
| Fig S4D 4X HFS  AAV9-GFP v.s. AAV9-Cre | -10-0 min  15-25 min  65-75min | Fisher's LSD *post hoc* test | 0.838  0.204  0.954 |
| Fig S4D 4X HFS  AAV9-GFP v.s. AAV8-GFP | -10-0 min  15-25 min  65-75min | Fisher's LSD *post hoc* test | 0.922  0.390  0.175 |
| Fig S4D 4X HFS  AAV9-GFP v.s. cWT+AAV9-Cre | -10-0 min  15-25 min  65-75min | Fisher's LSD *post hoc* test | 0.807  < 0.001 ***  < 0.001 *** |
| Fig S4D 4X HFS  AAV9-Cre v.s. AAV8-GFP | -10-0 min  15-25 min  65-75min | Fisher's LSD *post hoc* test | 0.921  0.697  0.224 |
| Fig S4D 4X HFS  AAV9-Cre v.s. cWT+AAV9-Cre | -10-0 min  15-25 min  65-75min | Fisher's LSD *post hoc* test | 0.998  0.006 **  < 0.001 *** |
| Fig S4D 4X HFS  AAV8-GFP v.s. cWT+AAV9-Cre | -10-0 min  15-25 min  65-75min | Fisher's LSD *post hoc* test | 0.906  0.002 **  0.012 * |
| Fig S5A  RIP-qPCR | *Gapdh* v.s. *Slc17a7*  *Gapdh* v.s. *Slc17a6*  *Gapdh* v.s. *Syt1* | t-test | 0.912  0.0281 *  0.0125 * |
| Fig S5B  cWT v.s. cKO^Nes^ | VGLUT1  VGLUT2  SYT1  CPEB2 | t-test | 0.937  0.00123 **  0.590  0.0114 * |
| Fig S5B  cWT v.s. cKO^Vglut2^ | VGLUT1  VGLUT2  SYT1  CPEB2 | t-test | 0.641  0.00172 **  0.871  0.0159 * |
| Fig S6  VGLUT2 | genotype  min | Two-way ANOVA  F_(1,84)_ = 15.927  F_(5,84)_ = 2.330 | < 0.001 ***  0.049 * |
| Fig S6  VGLUT2  CP2-WT v.s. CP2-KO | Ctrl  0’  15’  30’  60’  120’ | Fisher's LSD *post hoc* test | 1.000  0.010 *  0.077  0.091  0.057  0.089 |
| Fig S6  VGLUT2  CP2-WT | Ctrl v.s. 0’  Ctrl v.s. 15’  Ctrl v.s. 30’  Ctrl v.s. 60’  Ctrl v.s. 120’  0’ v.s. 15’  0’ v.s. 30’  0’ v.s. 60’  0’ v.s. 120’  15’ v.s. 30’  15’ v.s. 60’  15’ v.s. 120’  30’ v.s. 60’  30’ v.s. 120’  60’ v.s. 120’ | Fisher's LSD *post hoc* test | 0.002 **  0.011 *  0.011 *  0.005 **  0.083  0.567  0.568  0.804  0.163  0.999  0.745  0.406  0.746  0.406  0.249 |
| Fig S6  VGLUT2  CP2-KO | Ctrl v.s. 0’  Ctrl v.s. 15’  Ctrl v.s. 30’  Ctrl v.s. 60’  Ctrl v.s. 120’  0’ v.s. 15’  0’ v.s. 30’  0’ v.s. 60’  0’ v.s. 120’  15’ v.s. 30’  15’ v.s. 60’  15’ v.s. 120’  30’ v.s. 60’  30’ v.s. 120’  60’ v.s. 120’ | Fisher's LSD *post hoc* test | 0.588  0.427  0.380  0.328  0.970  0.800  0.736  0.662  0.614  0.933  0.854  0.449  0.920  0.401  0.347 |
| Fig S6  LRP130 | genotype  min | Two-way ANOVA  F_(1,84)_ = 0.0718  F_(5,84)_ = 0.149 | 0.789  0.980 |
| Fig S6  p-CaMK2α | genotype  min | Two-way ANOVA  F_(1,84)_ = 0.672  F_(5,84)_ = 3.960 | 0.415  0.003 ** |
| Fig S6  p-CaMK2α  CP2-WT v.s. CP2-KO | Ctrl  0’  15’  30’  60’  120’ | Fisher's LSD *post hoc* test | 1.000  0.720  0.481  0.721  0.856  0.690 |
| Fig S6  p-CaMK2α  CP2-WT | Ctrl v.s. 0’  Ctrl v.s. 15’  Ctrl v.s. 30’  Ctrl v.s. 60’  Ctrl v.s. 120’  0’ v.s. 15’  0’ v.s. 30’  0’ v.s. 60’  0’ v.s. 120’  15’ v.s. 30’  15’ v.s. 60’  15’ v.s. 120’  30’ v.s. 60’  30’ v.s. 120’  60’ v.s. 120’ | Fisher's LSD *post hoc* test | 0.481  0.004 **  0.117  0.487  0.772  0.025 *  0.383  0.992  0.677  0.164  0.025 *  0.008 **  0.377  0.199  0.685 |
| Fig S6  p-CaMK2α  CP2-KO | Ctrl v.s. 0’  Ctrl v.s. 15’  Ctrl v.s. 30’  Ctrl v.s. 60’  Ctrl v.s. 120’  0’ v.s. 15’  0’ v.s. 30’  0’ v.s. 60’  0’ v.s. 120’  15’ v.s. 30’  15’ v.s. 60’  15’ v.s. 120’  30’ v.s. 60’  30’ v.s. 120’  60’ v.s. 120’ | Fisher's LSD *post hoc* test | 0.728  0.025 *  0.223  0.607  0.913  0.057  0.382  0.868  0.648  0.295  0.081  0.019 *  0.479  0.185  0.533 |
| Fig S6  CaMK2α | genotype  min | Two-way ANOVA  F_(1,84)_ = 0.0726  F_(5,84)_ = 0.694 | 0.788  0.629 |
| Fig S8 | hour  protein | Two-way ANOVA  F_(7,80)_ = 4.654  F_(5,80)_ = 8.038 | < 0.001 ***  < 0.001 *** |
| Fig S8  Protein | VGLUT2 v.s. SYT1  VGLUT2 v.s. LRP130  VGLUT2 v.s. CPEB2  VGLUT2 v.s. VGLUT1  VGLUT2 v.s. GAPDH  SYT1 v.s. LRP130  SYT1 v.s. CPEB2  SYT1 v.s. VGLUT1  SYT1 v.s. GAPDH  LRP130 v.s. CPEB2  LRP130 v.s. VGLUT1  LRP130 v.s. GAPDH  CPEB2 v.s. VGLUT1  CPEB2 v.s. GAPDH  VGLUT1 v.s. GAPDH | Fisher's LSD *post hoc* test | < 0.001 ***  0.001 **  0.338  0.001 **  < 0.001 ***  0.132  < 0.001 ***  0.039 *  0.167  0.014 *  0.721  0.909  0.019 *  0.010 *  0.629 |
| Fig S8  VGLUT2 | 0 v.s.0.5  0 v.s.1  0 v.s.2  0 v.s.4  0 v.s.8  0 v.s.16  0 v.s.24  0.5 v.s.1  0.5 v.s.2  0.5 v.s.4  0.5 v.s.8  0.5 v.s.16  0.5 v.s.24  1 v.s.2  1 v.s.4  1 v.s.8  1 v.s.16  1 v.s.24  2 v.s.4  2 v.s.8  2 v.s.16  2 v.s.24  4 v.s.8  4 v.s.16  4 v.s.24  8 v.s.16  8 v.s.24  16 v.s.24 | Fisher's LSD *post hoc* test | 0.300  0.160  0.185  0.009 **  0.017 *  0.005 **  0.002 **  0.707  0.770  0.108  0.164  0.070  0.028 *  0.933  0.215  0.307  0.148  0.067  0.186  0.270  0.126  0.056  0.825  0.833  0.547  0.666  0.411  0.695 |
| Fig S8  SYT1 | 0 v.s.0.5  0 v.s.1  0 v.s.2  0 v.s.4  0 v.s.8  0 v.s.16  0 v.s.24  0.5 v.s.1  0.5 v.s.2  0.5 v.s.4  0.5 v.s.8  0.5 v.s.16  0.5 v.s.24  1 v.s.2  1 v.s.4  1 v.s.8  1 v.s.16  1 v.s.24  2 v.s.4  2 v.s.8  2 v.s.16  2 v.s.24  4 v.s.8  4 v.s.16  4 v.s.24  8 v.s.16  8 v.s.24  16 v.s.24 | Fisher's LSD *post hoc* test | 0.727  0.820  0.758  0.944  0.708  0.818  0.480  0.903  0.967  0.780  0.980  0.563  0.292  0.935  0.875  0.883  0.648  0.351  0.812  0.947  0.591  0.311  0.760  0.764  0.438  0.546  0.281  0.634 |
| Fig S8  LRP130 | 0 v.s.0.5  0 v.s.1  0 v.s.2  0 v.s.4  0 v.s.8  0 v.s.16  0 v.s.24  0.5 v.s.1  0.5 v.s.2  0.5 v.s.4  0.5 v.s.8  0.5 v.s.16  0.5 v.s.24  1 v.s.2  1 v.s.4  1 v.s.8  1 v.s.16  1 v.s.24  2 v.s.4  2 v.s.8  2 v.s.16  2 v.s.24  4 v.s.8  4 v.s.16  4 v.s.24  8 v.s.16  8 v.s.24  16 v.s.24 | Fisher's LSD *post hoc* test | 0.970  0.952  0.941  0.998  0.245  0.273  0.206  0.921  0.971  0.972  0.261  0.290  0.220  0.893  0.949  0.222  0.248  0.186  0.943  0.276  0.306  0.234  0.246  0.274  0.207  0.947  0.919  0.866 |
| Fig S8  CPEB2 | 0 v.s.0.5  0 v.s.1  0 v.s.2  0 v.s.4  0 v.s.8  0 v.s.16  0 v.s.24  0.5 v.s.1  0.5 v.s.2  0.5 v.s.4  0.5 v.s.8  0.5 v.s.16  0.5 v.s.24  1 v.s.2  1 v.s.4  1 v.s.8  1 v.s.16  1 v.s.24  2 v.s.4  2 v.s.8  2 v.s.16  2 v.s.24  4 v.s.8  4 v.s.16  4 v.s.24  8 v.s.16  8 v.s.24  16 v.s.24 | Fisher's LSD *post hoc* test | 0.458  0.219  0.279  0.053  0.049 *  0.015 *  0.006 **  0.623  0.732  0.228  0.213  0.084  0.043 *  0.882  0.472  0.448  0.213  0.121  0.386  0.365  0.164  0.090  0.968  0.595  0.400  0.623  0.423  0.755 |
| Fig S8  VGLUT1 | 0 v.s.0.5  0 v.s.1  0 v.s.2  0 v.s.4  0 v.s.8  0 v.s.16  0 v.s.24  0.5 v.s.1  0.5 v.s.2  0.5 v.s.4  0.5 v.s.8  0.5 v.s.16  0.5 v.s.24  1 v.s.2  1 v.s.4  1 v.s.8  1 v.s.16  1 v.s.24  2 v.s.4  2 v.s.8  2 v.s.16  2 v.s.24  4 v.s.8  4 v.s.16  4 v.s.24  8 v.s.16  8 v.s.24  16 v.s.24 | Fisher's LSD *post hoc* test | 0.903  0.698  0.665  0.670  0.246  0.148  0.077  0.610  0.579  0.584  0.201  0.118  0.060  0.964  0.969  0.439  0.288  0.165  0.995  0.465  0.309  0.179  0.462  0.306  0.177  0.772  0.536  0.742 |
| Fig S8  GAPDH | 0 v.s.0.5  0 v.s.1  0 v.s.2  0 v.s.4  0 v.s.8  0 v.s.16  0 v.s.24  0.5 v.s.1  0.5 v.s.2  0.5 v.s.4  0.5 v.s.8  0.5 v.s.16  0.5 v.s.24  1 v.s.2  1 v.s.4  1 v.s.8  1 v.s.16  1 v.s.24  2 v.s.4  2 v.s.8  2 v.s.16  2 v.s.24  4 v.s.8  4 v.s.16  4 v.s.24  8 v.s.16  8 v.s.24  16 v.s.24 | Fisher's LSD *post hoc* test | 0.976  0.911  0.860  0.640  0.452  0.395  0.351  0.887  0.836  0.619  0.434  0.379  0.335  0.948  0.722  0.521  0.460  0.411  0.771  0.564  0.500  0.449  0.775  0.701  0.640  0.922  0.856  0.933 |
